# Modeling and simulation of dual-battery FANET system using MADRL for energy optimization

**DOI:** 10.1038/s41598-025-30429-z

**Published:** 2025-12-19

**Authors:** A. Sophia Mary, T. S. Pradeep Kumar

**Affiliations:** 1 School of Electronics Engineering, Vellore Institute of Technology, Chennai, 600127 India; 2 School of Computer Science and Engineering, Vellore Institute of Technology, Chennai, 600127 India

**Keywords:** Electrical and electronic engineering, Computer science

## Abstract

Uncrewed Aerial Vehicles (UAVs), or drones, are revolutionizing applications such as surveillance, search and rescue (SAR), precision agriculture, and disaster response. In these applications, Flying Ad-Hoc Networks (FANETs)—dynamic networks of interconnected UAVs—are essential for enabling rapid and adaptive deployments. However, the limited battery life of UAVs remains a critical challenge, particularly in extended missions like SAR, where operational longevity and reliable performance are paramount. This paper addresses two key challenges in FANET-based UAV operations: optimizing energy consumption and ensuring stable communication links. We propose a novel strategy that models energy-efficient UAV behavior by focusing on the three primary drivers of power consumption: flight dynamics, payload operations, and continuous wireless communication, which collectively account for 80–85% of the UAV’s energy usage. Leveraging a multi-agent deep reinforcement learning (MADRL) framework that intelligently manages a dedicated dual-battery system: one battery (B1) optimized for flight dynamics and payload operations, and the other (B2) for processor/sensor and continuous wireless communication. The MADRL agents (Proximal Policy Optimization: PPO) are modeled to handle battery B2, which dynamically establishes communication between UAVs regarding energy usage and other environmental conditions during SAR operations, thereby efficiently conserving available energy to extend the battery life of each UAV within the FANET system. Experimental results demonstrate that MADRL enhances network connectivity and significantly reduces energy wastage, enabling sustained operations over longer durations. This research lays the foundation for developing energy-efficient UAV systems, which are crucial for large-scale and autonomous deployments in mission-critical scenarios.

## Introduction

**Fig. 1:**
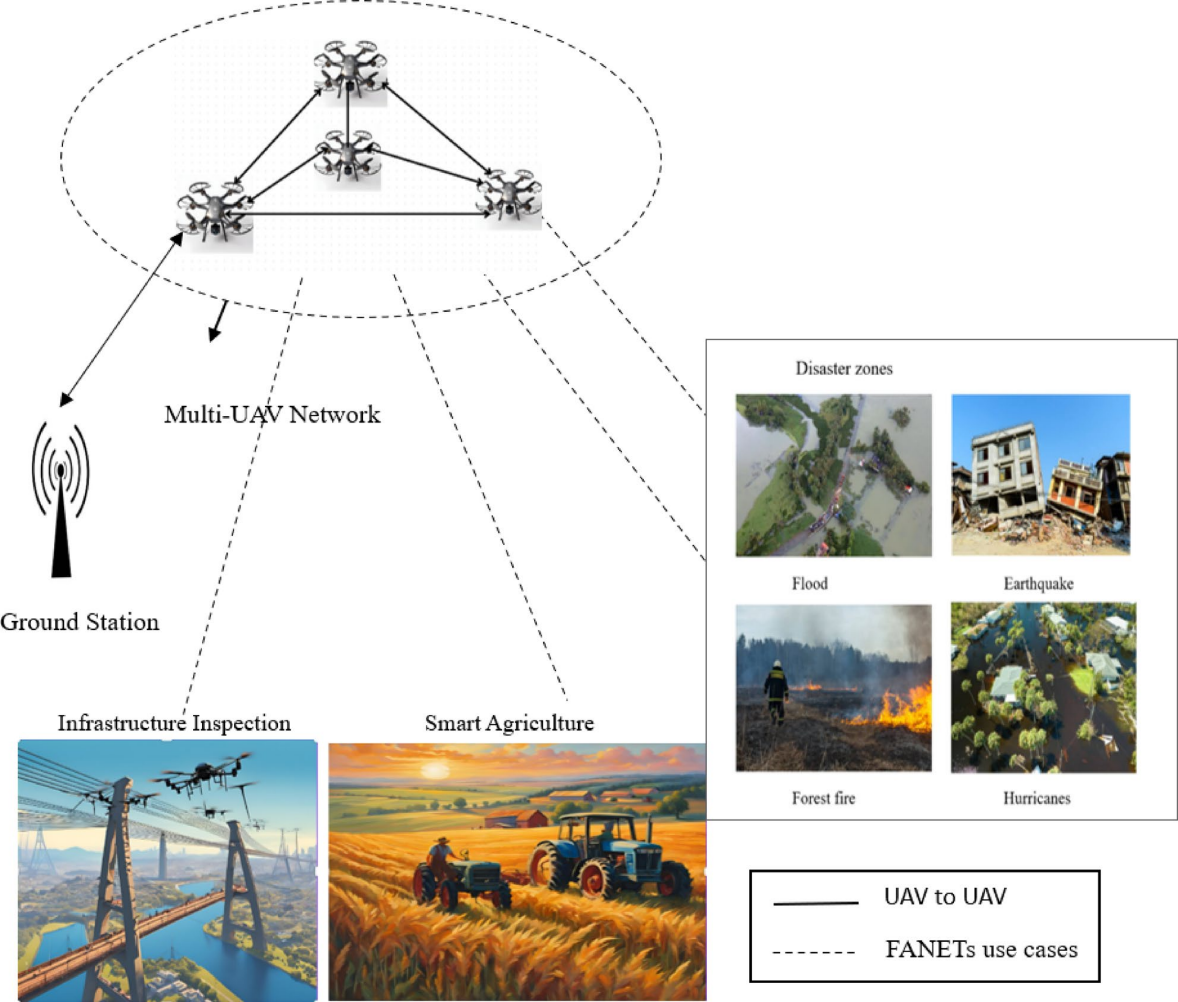
FANETs in UAV applications—Distributed Architecture. The image in Fig. 1 was created using Canva (https://www.canva.com).

The rapid evolution of UAVs has transformed the landscape of modern aerial robotics, enabling innovative solutions across industries such as public safety, environmental monitoring, agriculture, and defense^1–5^. The battery is a key component of UAVs, powering the applications in these fields. These battery-powered autonomous or remotely operated systems are equipped with advanced flight software, onboard sensors, cameras, and GPS technology, allowing them to execute complex tasks with high precision^6^. Initially developed for military purposes only, for dropping bombs on the battlefield, surveillance, and reconnaissance^7^, UAVs have now become indispensable in civilian applications as shown in Fig. 1, including SAR missions, disaster response, agriculture, delivery, and infrastructure inspections. The important abbreviations and notations are summarized in Table 1.

**Table 1:** Summary of abbreviations and important notations used in this work.

Abbreviations	Description	Notation	Description
UAV	Uncrewed Aerial Vehicles	S_t_​	State of the UAV at time step t
B1	Battery for flight dynamics	S_t_​_+1_	Next state of the UAV after action At A_t At​
B2	Battery for Communication	A_t_	Action taken by the UAV at time step t
MADRL	Multi-Agent Deep Reinforcement Learning	R_t_	Reward received at time step t
PPO	Proximal Policy Optimization	$\:{\pi\:}_{\theta\:}$	Policy parameterized by θ, mapping states to actions
SAR	Search and Rescue	θ	Parameters of the policy network
GPS	Global Position System	Ύ	Discount factor
QoS	Quality of Service	π*,	Optimal policy for UAV behaviour
RL	Reinforcement Learning	$\:{E}_{{\pi\:}_{\theta\:}}$​​	Expectation over states and actions under policy πθ
BMS	Battery Management System	∇_θ_​R_t_​	Gradient of the expected return with respect to θ
EOD	End of Discharge	π_θ_​(a_t_​∥s_t_​;θ)	Policy probability distribution over actions
SOC	State of Charge	∇θ​logπθ​(at​∥st​;θ)	Gradient of the log probability of action a_t_​
BHM	Battery Health Management	A​	Advantage function estimating action quality
EMS	Energy Management System	Q(St​,At​)	Action-value function
FEM	Finite Element Model	∇θ​logπθ​(at​∥st​;θ)	Gradient of the log probability of action a_t_​
ECMs	Equivalent Circuit Models	V(St​;ϕ)	State-value function with weights ϕ
NS3	Network Siulator	δt​	Temporal difference (TD) error
MDP	Markov Decision Problems	λ	GAE parameter for advantage estimation
ECMs	Electrochemical Models	ϕ	Parameters of the value function network
MlpPolicy	Multi-layer perceptron	LPG​(θ)	Policy gradient objective function
TCP	Transmision Control Protocol	r_t_​(θ)	Probability ratio between current and old policy
α	Learning rate	E_B1_​	Energy of the primary battery (B1)
c1​,c2​	Weights for value function loss and entropy bonus	E_B1_​	Energy of the secondary battery (B2)
π_θ_old​	Policy parameters before update	T_E_	Total energy (EB1 + EB2​)

With the growing reliance on UAVs for critical, large-scale operations, researchers have introduced multi-UAV systems, often called UAV swarms or FANETs, to enhance operational efficiency^8^. FANETs consist of multiple UAVs working collaboratively within a distributed network, enabling real-time data sharing, improved coverage, and autonomous mission execution. These systems are particularly valuable in dynamic environments, such as SAR operations^9,10^, which is the primary focus of this paper.

For instance, coordinated multi-UAVs can autonomously patrol and monitor urban areas, supporting law enforcement in maintaining public safety. They can cover large areas, identify potential threats, and assist in real-time law enforcement operations^11^. This technological advancement proves especially beneficial in scenarios where human intervention may be limited or unsafe, such as post-disaster reconnaissance, wildfire surveillance, and large-scale environmental monitoring^11^.

Despite their growing deployment, battery-powered UAVs face a major limitation—short flight endurance due to the low energy density of current battery technologies^12,13^. Energy-efficient UAV mission planning is crucial to conserve available energy, as high-speed mobility, continuous data transmission, and environmental resistance accelerate battery depletion. Addressing energy efficiency remains a key research challenge for enhancing UAV performance. In FANETs, power exhaustion of even a single UAV can disrupt network communication, degrade mission performance, and delay critical decision-making processes^14^. This limitation is particularly problematic in life-saving scenarios, such as SAR missions, where uninterrupted aerial monitoring is crucial for identifying survivors and assessing disaster zones (Fig. 1).

**Unique challenges of UAVs in energy modelling**

Despite their versatility and growing adoption, UAVs face significant design challenges, with limited flight time being one of the most critical constraints. This limitation primarily arises from two key factors:

1. High Energy Demand – UAVs necessitate a considerable amount of power to support critical functions such as hovering, high-speed flight, stability control, and real-time data processing.
2. Battery Capacity Constraints – UAVs rely on onboard batteries with limited energy storage, restricting their ability to operate over large areas or for extended durations^15,16^.

To address these challenges and enhance cruising time, smart energy management strategies are essential. Predicting battery status, specifically remaining energy, is vital for energy-efficient mission planning, ensuring both optimal performance and battery longevity. An efficient energy consumption model underpins this effort by estimating flight time, predicting energy usage across diverse operational demands, and optimizing power consumption. Developing accurate energy models and energy-efficient UAV architectures is key to managing energy effectively, enabling longer flight durations, seamless FANET operations, and robust performance in real-world applications. This research aims to minimize energy waste in UAVs and extend their flight time by utilizing advanced energy modeling, dual-battery resource allocation, and optimization techniques through MADRL. By applying RL technology to a FANET, UAVs (agents) can automatically learn and adapt to dynamic network characteristics, such as fluctuating link quality, frequent changes in network topology, and varying energy consumption.

This approach addresses challenges that conventional network technologies struggle to handle, ultimately enhancing network performance and improving Quality of Service (QoS). However, RL faces challenges in complex network scenarios, such as FANETs in SAR environments. The large number of nodes and the constantly changing environment make it difficult to maintain an extensive Q-table, resulting in significant storage requirements and time overhead. Furthermore, RL struggles to scale effectively to larger state and action spaces, limiting its applicability in large-scale, dynamic settings. To address these limitations, we combine RL with deep learning techniques. Deep Reinforcement Learning (DRL) has made significant advancements in autonomous decision-making and intelligent control, offering promising solutions to handle the complexity and scalability issues in such environments. In this work, we employ a PPO-based energy optimization technique, where an energy policy is a mapping from action space to state space for energy-efficient SAR mission operation.

## Background and motivations

**Fig. 2:**
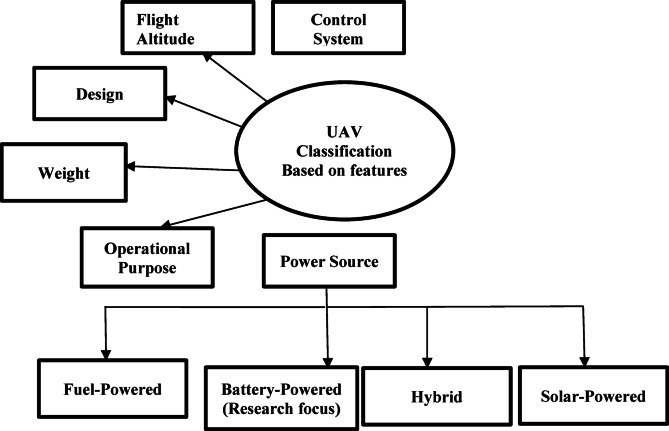
UAV classification on unique features.

Battery-powered UAVs are becoming increasingly popular and are dominating the UAV market due to their high operational efficiency, lower operational cost, and ease of use. As of 2023, they account for approximately 90% of the market share, making them the preferred choice for a wide range of aerial missions^17^. UAVs are typically classified based on various factors, including weight, design, flight altitude, control system, operational purpose, take-off and landing mechanisms, and power propulsion systems^18^ as depicted in Fig. 2. Based on their power sources, UAVs can be categorized into five types: battery-powered, fuel-powered, hybrid, and solar-powered UAVs, as shown in Fig. 2.

Although UAVs in the past primarily used fuel as a power source due to its high energy density, this power source is environmentally harmful^19,20^. Hybrid-powered UAVs use a combination of batteries and fuel for extended endurance^21^. While they are primarily used in electric vehicles, their application in the UAV industry remains limited. Because the effectiveness of these UAVs depends on a highly efficient energy management system (EMS). Unfortunately, the strategies for implementing this EMS in UAVs are still developing^22^. In contrast, battery-powered UAVs operate entirely on electric energy, resulting in zero emissions and supporting green technology initiatives. These UAVs are simple, lightweight, and cost-effective, making them the mainstream choice in various applications. The most commonly used battery type on UAVs is lithium-ion. Batteries of this type have low energy density, are short-lived, and last only 20–40 min. However, they have high power density, do not emit polluting substances, and are perfect for UAV applications.

The battery is a critical component that directly impacts UAV performance, endurance, and overall mission success. For instance, battery-powered autonomous multi-UAV systems are widely used in SAR missions, offering real-time situational awareness, large-area coverage, and the ability to operate in hazardous environments where human responders face risks. However, these UAVs face significant energy limitations due to continuous real-time monitoring, constant data transmission, and payload-carrying, all of which lead to rapid energy depletion. If a UAV’s battery runs out mid-mission, it can be catastrophic—limiting endurance, disrupting the mission, or even causing mission failure, potentially compromising critical operations. To lessen the impact of limited battery capacity, it is essential to reduce energy waste and implement optimized power allocation strategies in UAV operations. For instance, if the model predicts B2 will deplete mid-mission due to heavy FANET communication, the MADRL can pre-emptively scale back non-essential links. This intelligence extends cruising time and boosts longevity by avoiding overstress on batteries.

To enable energy-efficient UAV operations and minimize energy consumption across various UAV activities—such as flight manoeuvring, communication, and payload operations—we need reliable and comprehensive energy consumption modeling that understands the battery status and optimized power allocation strategies rather than relying solely on the Battery Management System (BMS).

Most existing research on BMS primarily addresses battery protection, charging, and monitoring. However, BMS lacks the predictive capabilities necessary to dynamically optimize the endurance of UAVs. In contrast, energy consumption modeling plays a vital role in enhancing BMS by analysing energy consumption patterns and predicting battery discharge behavior, such as estimating the remaining energy and End of Discharge (EOD). This area remains largely underexplored in UAV research, which is the focus of this work.

Developing an accurate energy consumption and dual battery model can significantly enhance UAV endurance, optimize power allocation, and improve mission success rates by addressing different aspects of power management. Motivated by these challenges, we propose an intelligent dual-battery UAV system model specifically tailored for FANETs (multi-agent) environments like SAR operations. This model profiles energy demand across diverse operational conditions, including flight manoeuvring, mobility, communication, and payload, ensuring efficient energy utilization and extended mission capability. The model for this work uses a mathematical modelling approach to analyse the behavior of a dual battery system in a UAV, estimating its performance, energy consumption patterns, and operational characteristics of UAV operation.

The core concept is to leverage two batteries, Battery 1 (B1) and Battery 2 (B2), with a functional segmentation: B1 primarily powers flight dynamics, mobility, and propulsion, while B2 supports communication and payload operations. Hence, these two separate power sources for flight (main battery) and communication (small battery) isolate critical functions and prevent one system from draining the other. The intelligence (MADRL) applied to B2 learns to balance energy usage and communication needs, adapting to each other’s status and the environment without impacting B1 energy. By keeping the two batteries isolated, we can prevent a single point of failure and ensure that even if B2 depletes due to heavy communication demands, the UAV can still fly and return safely using B1.

The MADRL agent governing B2 analyzes real-time data, including its own State of Charge (SoC), the statuses of other UAVs, and mission demands, learning to balance energy usage with communication needs within the FANET. This intelligent management keeps the network informed without burdening B1, mitigating risks of rapid B2 depletion and mid-mission failures. Fundamentally, the dual-battery system isolates critical functions—B1 for flight and B2 for communication—while MADRL optimizes B2 to adapt to the FANET’s dynamic requirements and environmental conditions. This synergy preserves B1 for flight stability, extending mission duration and enhancing the UAV system’s reliability and efficiency in complex operational scenarios.

To validate this approach under realistic conditions, the research employs the open-source network simulator NS3 to model multi-UAV network environments, specifically Flying Ad-hoc Networks (FANETs). NS3 enables detailed simulation of a dual-battery system—B1 dedicated to flight dynamics and B2 for communication—capturing energy usage patterns and evaluating the effectiveness of Multi-Agent Deep Reinforcement Learning (MADRL) applied to B2. By simulating real-world networking scenarios, including varying environmental conditions and battery characteristics, NS3 ensures the practicality and robustness of the proposed system. The MADRL agents, guided by real-time B2 status and inter-UAV coordination data, learn to balance communication needs with energy conservation, optimizing B2 usage without compromising B1’s reserves. This strategy preserves B1 for flight stability, enhancing FANET endurance, while B2’s intelligent management prevents premature depletion, ensuring reliable, continuous operation. The result is a comprehensive power management solution that combines the isolation benefits of a dual-battery design with MADRL-driven decision-making, significantly improving mission success rates in complex operational environments. Table 2 illustrates the advantages of this dual-battery system, underscoring its contributions to prolonged UAV endurance and enhanced mission outcomes.

**Table 2:** Proposed dual battery VS existing single battery system in UAV.

Feature	Single-Battery System	Dual-Battery System (Proposed)	Reasons
Energy Efficiency	✖	☑	Dual-battery system for better energy distribution.
Flight Endurance	✖	☑	Dual-battery system increases overall flight duration.
Mission Reliability	✖	☑	Dual-battery system reduces the risk of mid-mission failure.
Power Management (Intelligent)	✖	☑	Dual-battery system allows for dynamic power allocation.
Complexity and Cost	☑	✖	Single-battery systems are simpler and cheaper to implement.

### Contributions and novel findings of the proposed study

In this research, we focus on advancing UAV battery modeling and energy optimization by addressing limitations in existing approaches. Our key contributions and new findings are:

1. Introduction of a Dual-Battery Model for FANET(Multi-UAV) Systems in SAR operation:

We propose a novel dual-battery UAV system, where:

- The first battery B1 handles propulsion and flight manoeuvring.
- The second battery, B2, manages system power, communication, and sensors.

By this approach, we can achieve dynamic energy balancing (based on UAV workload), extending UAV endurance and ensuring uninterrupted mission execution in SAR operations.

2. Mathematical Battery Modelling for UAV Energy Consumption.

We develop an analytical energy consumption model considering UAV power demands for flight manoeuvring (propulsion, mobility), communication (Tx/Rx), and process/sensor operations. By mathematically representing the UAV battery’s internal behavior, we can understand how power demands impact battery life and optimize energy use to improve capacity. This approach also reduces computational cost and enables faster, safer testing through simulation.

3. Simulation and Validation Using NS3:

We implement the proposed model in the NS3 network simulator to evaluate the FANET system’s battery status, energy consumption, and overall performance. This simulation helps validate the effectiveness of our dual-battery model in optimizing energy usage and extending the UAV’s mission duration.

4. Intelligence applied to Secondary Battery B2 using Deep Reinforcement Learning (DRL) and simulating in the NS3-Gym framework.

We employ DRL for predictive decision-making in energy allocation and control of UAV states based on real-time conditions such as SoC or remaining energy in the battery, power demand, and mission constraints. Furthermore, to train and validate the DRL agent’s performance in managing Battery B2, we utilize the ns3-gym framework. This framework allows the DRL agent to interact with the simulated NS3 network environment, learning optimal energy management policies through observation of network states, execution of actions, and reception of corresponding rewards. By integrating NS3 and ns3-gym, we can comprehensively evaluate both the dual-battery system’s efficacy and the DRL agent’s ability to adapt to complex FANET conditions dynamically.

The remainder of this article is structured as follows: The section “Background and motivations” provides the background and motivation for this work. The section “Related works“ gives a deep insight into earlier attempts at modeling the energy consumption and battery of UAVs. The section “Modelling energy consumption of UAV dual battery system” presents and discusses the proposed energy consumption modeling for this work; the section “Proposed DRL-based energy optimization for FANET system in SAR operation” presents and discusses the proposed DRL methodology for battery energy optimization. The section “Experimental evaluation and results discussion” focuses on the experimental evaluation and the discussion of results. Finally, the section “Conclusion” concludes the article and outlines potential future work.

## Related works

UAVs are now a mandatory practice in disaster zones, as illustrated in Fig. 1. In such environments, UAVs are important because they can provide real-time information, enhance search and rescue operations by keeping human responders safe, assess damage quickly, and offer a cost-effective and flexible alternative to traditional manned aircraft and ground vehicles^23–25^. However, one major limitation is that onboard batteries are often not large enough to support most UAV functions, especially during search and rescue missions. Due to design constraints, UAVs are typically unsuitable for continuous and long-term monitoring, which necessitates robust battery performance estimation models to prevent mid-mission failures.

Even with FANET technology, where multiple UAVs collaborate on SAR missions to expand coverage, each UAV still has a limited battery life. When a UAV’s battery is depleted, it must either be returned to a charging station or replaced. This disrupts the network and forces other UAVs to use extra energy to find and reconnect with a new neighbour node, which affects overall efficiency. In response to these challenges, researchers are exploring various solutions, including energy-efficient path planning, implementing wireless charging systems, developing battery replacement methods, BMS, BHM (Battery Health Management), and the integration of solar panels and other energy-harvesting techniques.

In contrast to electric vehicles, which have been extensively studied for energy consumption modeling, most research has concentrated on ground vehicles^26–30^. UAVs, however, face unique power constraints due to their reliance on battery-powered propulsion, continuous communication, and ongoing sensor operations. Unlike ground vehicles, UAVs experience dynamic energy consumption that is influenced by varying factors, including flight dynamics (such as propulsion and mobility), changes in altitude, payload variations, and environmental conditions. As a result, this research aims to develop an energy model specifically for UAV applications and improve battery performance, ensuring better efficiency and reliability in real-world missions.

E. Kim et al.^31^ emphasize the importance of predicting battery life for autonomous battery management systems (BMS) based on energy consumption during UAV missions. Zhang et al.^32^ investigated the impact damage of lithium batteries in small UAVs using experimental and simulation methods. They developed and validated a finite element model (FEM) through impact compression tests, which demonstrated minimal errors (≤ 5.67%). The findings revealed that the external battery box becomes less effective at high impact speeds, increasing the risk of internal battery damage, explosion, and fire. This underscores the necessity for impact-resistant battery enclosures in UAVs.

The authors in^33^ address the energy limitations of multi-UAV networks in search and rescue (SAR) missions by proposing a mission-planning method that optimizes UAV trajectories, recharging schedules, and charging station placements to minimize mission completion time while ensuring efficient energy use. Zhang et al.^34^ review and classify various drone energy consumption models, analyzing their scope, design factors, and operational assumptions. They highlight that most studies rely on simplified, often linear, models that overlook nonlinear wind effects, which can result in energy predictions up to two times lower than actual consumption. Their findings also emphasize the wide variability in energy predictions across models and underscore the need for empirical validation to improve accuracy. The authors in^35^ propose a Bayesian inference-based battery health management technique to manage the energy consumption in UAVs.

Battery modeling can be classified into three main types: Equivalent Circuit Models (ECMs), Electrochemical Models (EMs), and Data-driven (Black Box) Models. Several studies highlight that constant resistance battery models are well-suited for UAV applications due to their simplicity and efficiency^36–39^. These models provide a straightforward electrical representation, assuming constant internal resistance during operation, making them useful for basic energy management. However, their lack of adaptability to dynamic flight conditions and inability to model long-term battery degradation limit their effectiveness in real-world UAV missions.

**Fig. 3:**
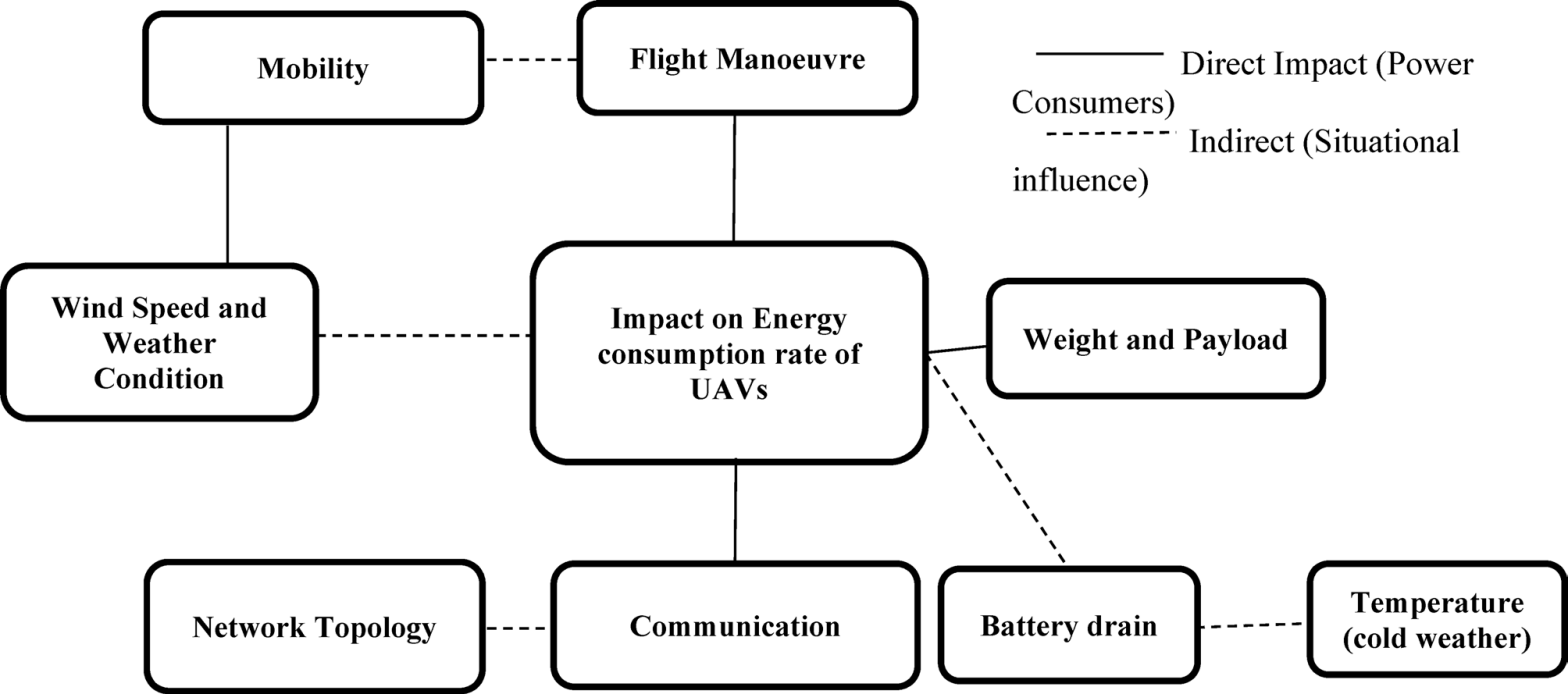
Main aspects of the energy consumption rate for FANET operation.

The existing literature highlights a significant research gap in addressing limited battery life in UAVs. While most studies have explored the estimation of UAV energy consumption, they often fail to fully model real-time power demand fluctuations and battery degradation dynamics, both of which are crucial for enhancing efficiency and extending flight time. Moreover, research on UAV battery performance estimation remains limited. Hence, this research aims to develop a better battery model capable of accurately predicting and managing energy consumption across key operational aspects, as illustrated in Fig. 3. These aspects include flight manoeuvres, communication, and payload handling, all of which are critical to enhancing overall mission performance and success.

Dynamic battery energy management, which intelligently adjusts the power based on real-time demands, is presented as a potential solution for extending battery life. By tailoring energy use to different tasks, the system can reduce stress on the battery and maximize efficiency. Therefore, incorporating an accurate and reliable battery energy consumption model is crucial for time-sensitive FANET missions. Without it, missions could be compromised, leading to failures, unnecessary delays, and increased energy consumption, ultimately diminishing the efficiency and operational success of UAVs. Table 3 presents the overview of the current literature on energy consumption models, and Table 4 discuss the related works on DRL techniques used specifically for UAVs.

**Table 3:** Overview of recent literature on energy consumption modelling of UAV.

References	Problem	Energy Aspects considered	Modeling’s approach	Optimization Techniques	Key findings & Batter performance considered[Y/*N*]	Limitations
^40^	UAV swarm communication coverage optimization	Energy usage information change, connectivity restoration	Theoretical & ML-based models (XGBoost, regression)	Cost function for swarm topology control	Identified energy-efficient airspeed range; ML model achieved R² of 0.9999. Battery consideration: NO	Regression models lack extrapolation accuracy
^41^	Coverage path planning for UAVs	Weight and Maximum speed.	Energy model derived from real measurements	Energy-aware path planning algorithm	Minimizes energy consumption while ensuring coverage & resolution. Battery consideration: NO	Does not account for dynamic environmental conditions.
^42^	UAVs as mobile base stations for ground user coverage	Temporal energy consumption, connectivity, flight stability.	Deep neural network (DNN)-based model	Decentralized DRL framework (DDPG)	Improved coverage and fairness while reducing energy usage Battery consideration: NO	Requires extensive training data
^43^	Energy-efficient UAV target tracking	Velocity, altitude, acceleration impact on energy	Adaptive energy estimation model	Zone-based control for UAV adjustments	Limits UAV adjustments while maintaining target in view Battery consideration: NO	May not adapt well to sudden target movements
^44^	UAV-based last-mile delivery optimization	Energy consumption in relation to payload & battery weight	Mathematically derived energy model	Mixed-integer linear programming (MILP), Simulated Annealing (SA) heuristic	Demonstrates inverse exponential relationship between cost & delivery time Battery consideration: NO	Limited to theoretical and simulated validation
Proposed work	Energy-efficient UAV operations in FANETs for SAR missions	Flight dynamics, payload operations, wireless communication energy consumption	Mathematical analysis and simulation-based Multi-Agent Deep reinforcement learning (MADRL)	Dual-battery system design and intelligence applied to secondary battery.	Extends UAV endurance, maintains reliable connectivity, and improve overall performance. Battery consideration: Yes	To ensure cost-effectiveness and safety, NS3 simulations were used. Future work will focus on real-world implementation

**Table 4:** Related works explored DRL techniques in comparison with the proposed work.

References	Research Problem	Modelling approach	Key contributions	System/Vehicle/Device	Performance improvement
^45^	UAVs have limited battery life, affecting mission planning	Empirical energy model based on battery usage in different flight scenarios	Comprehensive UAV energy model. Accounts for flight conditions. Helps in energy-efficient planning.	UAVs	Better energy prediction for optimized missions
^46^	High power consumption in dense IoT deployments (e.g., smart city scenarios)	Green LoRa wireless networks powered by a hybrid of the grid and renewable energy sources	Proposes resource management schemes for channel and spreading factor (SF) allocation Formulates grid power consumption minimization problem Develop DRL-based adaptive resource management schemes Improves energy efficiency in LoRa networks	LoRa wireless networks	Efficient use of renewable energy and reduced grid power consumption
^47^	Resource management in massive IoT networks	Deep reinforcement learning (DRL) with UAV-based base stations and clustering	UAVs act as base stations for air-to-ground communication IoT devices are clustered into urban and suburban groups K-means and round-robin scheduling optimize resource allocation TensorFlow-based evaluation demonstrates the efficiency	UAV-based IoT networks	Rapid convergence, suitability for heterogeneous networks, and low complexity
Proposed work	Limited battery life and unstable communication in FANET-based UAV missions	MADRL with a dual-battery system, modelling, and simulation using NS3	Dual-battery system: B1 (flight/payload), B2 (communication/sensing), used our previous work hybrid 3-D Gauss Markov Mobility model, MADRL optimizes energy use and communication Enables energy-efficient dynamic UAV coordination in SAR	Multi-agent (FANET)	Improved network connectivity and reduced energy wastage for longer missions

In summary, numerous energy consumption models have been proposed for various UAV applications, employing diverse approaches to estimate power usage. However, these models often fail to accurately capture real-world UAV performance and energy efficiency, particularly in dynamic, multi-UAV environments like FANETs. As depicted in Fig. 3, energy consumption is predominantly driven by navigation, followed by communication, payload operations, and environmental factors such as wind or turbulence.

To address these challenges, this research proposes a robust energy prediction and management model featuring a dual-battery system: a primary battery (B1) dedicated to flight dynamics and an ultra-low-weight secondary battery (B2) for communication. Integrated with MADRL applied to B2, this approach enables real-time power management, optimizing energy usage and extending operational life without compromising flight stability or network connectivity. By isolating critical functions and dynamically adapting to FANET conditions, the proposed system ensures efficient UAV operation, sustained communication, and enhanced mission success in complex, real-world scenarios. While DRL has demonstrated remarkable success in various robotic and game-playing tasks, its application to complex communication system control, particularly in dynamic environments like FANETs, remains largely unexplored. Also, this research aims to bridge this gap, investigating the effectiveness of MADRL in optimizing energy management for UAV communication systems.

### Modelling energy consumption of UAV dual battery system

#### Problem statement

**Fig. 4:**
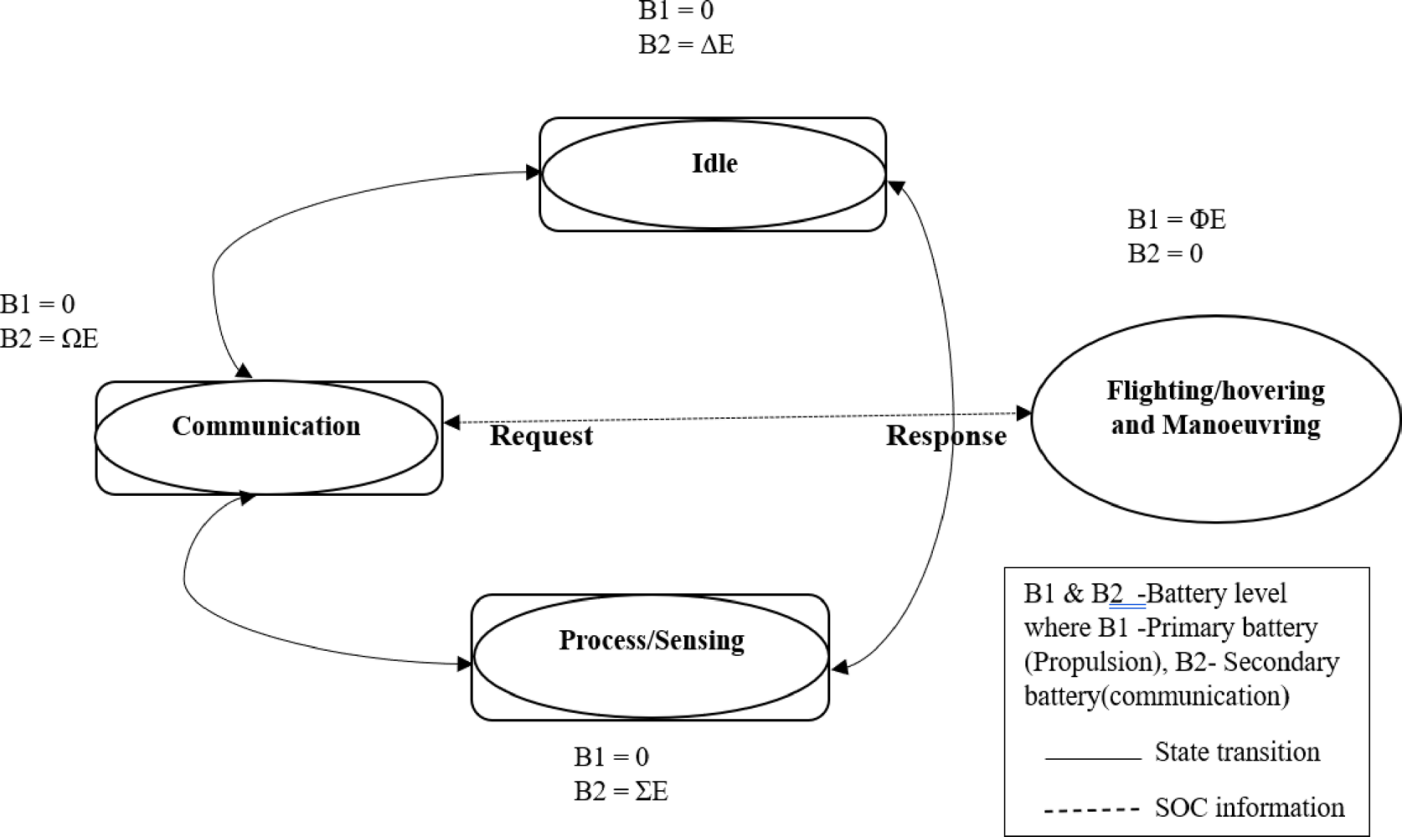
State Transition Diagram of a UAV in a FANET for SAR scene, illustrating energy consumption and battery energy management strategies.

The growing demand for electrical energy in UAV battery systems, especially for critical applications like SAR, highlights the need for accurate and reliable energy consumption modeling. A well-structured model can help optimize battery usage, enhance mission endurance, and improve UAV performance in real time. Before developing a comprehensive model, it’s crucial to identify and analyze the key factors that significantly influence energy consumption. This research focuses on the primary aspects of UAV power consumption, encompassing propulsion (motor operation), mobility, data transmission and reception, sensor usage, payload weight, and environmental conditions. Understanding how these factors contribute to battery drain is fundamental to creating an effective energy consumption model. Figure 4 illustrates the individual UAV system model within a FANET, depicting how energy is depleted under various operational conditions. As part of this work, we analyze the maximum energy consumption of hovering, high-speed flight, communication, and payload operations. Each scenario has unique power requirements due to its specific design, aerodynamics, and energy requirements. Therefore, we aim to model and simulate a dual-battery system in an NS3 simulator, where one battery handles mobility and propulsion while the other manages communication, processing, and sensing. Several modeling approaches have been proposed in the literature to maximize the battery life of UAVs. To optimize energy consumption, this work employs a DRL model based on Proximal Policy Optimization (PPO), which dynamically adjusts power allocation for communication and coordination tasks in FANET systems. Table 5 presents the energy consumption of UAVs in different operational modes, highlighting the impact of our proposed optimization technique.

**Table 5:** UAV operating States and energy demand.

UAV states	Battery usage	Description	Energy demand
Idle	B2	UAV is grounded or in a minimal power mode and is awaiting a mission start.	Low
Hovering	B1	UAV maintains position, requiring continuous thrust.	High
Flight manoeuvring	B1	Sudden change in speed, altitude, or direction.	Very high
Communication	B2	Data transmission and reception	Medium
Processing/Sensing	B2	Onboard computation and sensor operations.	Medium

#### Problem description

The high energy demands of UAVs coupled with their limited battery capacity present a significant challenge, particularly in FANET systems where sustained operation, continuous inter-UAV communication, and mobility are essential for mission success, as illustrated in Fig. 4. Navigation, encompassing propulsion and flight stability, dominates energy consumption, followed by communication for maintaining network connectivity, payload operations, and environmental influences such as wind or turbulence as shown in Table 5. Existing energy consumption models have sought to mitigate these constraints but often adopt a narrow focus—targeting specific elements like flight dynamics or environmental conditions—rather than holistically addressing the intricate interplay of all contributing factors as shown in Fig. 3. This fragmented approach hampers the development of a unified model capable of accurately reflecting the dynamic energy demands of FANET environments, resulting in suboptimal power allocation and diminished operational efficiency.

In FANETs, this challenge intensifies due to the persistent communication load required for real-time coordination among UAVs, which competes with the substantial energy needs of flight. Table 2 highlights the necessity of integrating an additional battery without increasing the overall UAV weight. Traditional single-battery architectures exacerbate the issue by coupling these critical functions—navigation and communication—leaving UAVs vulnerable to mid-mission failures when energy is depleted by excessive network demands or unforeseen environmental stressors. Furthermore, conventional static energy management strategies lack the flexibility to adapt to the diverse states, mission objectives, and environmental conditions across a UAV swarm, compromising flight stability, network reliability, and mission endurance. Consequently, these limitations undermine the effectiveness of UAVs in critical applications such as surveillance, disaster response, and environmental monitoring, where prolonged operation and robust connectivity are non-negotiable. This research underscores the urgent need for an innovative energy model and system design that dynamically optimizes power consumption, isolates flight and communication functions, and enhances energy efficiency to overcome these barriers and ensure successful FANET operations.

#### System model

The system model of the FANET, as illustrated in Fig. 4 and complemented by the depiction in Fig. 1, provides a comprehensive framework for optimizing UAV operations within a SAR mission in disaster-affected areas, with a strong emphasis on efficient energy management to enhance mission performance. The depiction in Fig. 1 highlights the application of the FANET system, where a network of UAVs collaboratively conducts search, surveillance, and target detection operations, operating as a distributed system with each UAV functioning as a mobile node to enable real-time communication and data sharing for effective coordination throughout the mission. This research aims to boost operational efficiency by maximizing communication performance, extending flight duration, and improving area coverage through strategic UAV energy expenditure. Figure 4 explains how UAVs operate through different states to save energy and complete their tasks effectively. Beginning with the idle state, where UAVs conserve energy while stationed or awaiting deployment, a low-power baseline is established to enhance endurance. From this state, UAVs transition to the process/sensing state, actively collecting data with sensors for surveillance and target detection, moderately increasing energy use to monitor the disaster environment. The process advances to the Flight manoeuvring state, which enables dynamic navigation to systematically cover the search area, reflecting the highest energy demand driven by propulsion requirements. At the model’s core, the Communication state facilitates real-time data sharing and coordination among UAVs, linking all states to ensure network reliability despite varying energy needs.

A key focus of this work is identifying the primary drivers of power consumption—propulsion energy, essential for flight and mobility, and communication energy, critical for network operations, recognizing that propulsion demands significantly outweigh communication needs. To address this, the research introduces an innovative dual-battery system, prioritizing propulsion with the primary battery while applying intelligent energy management to the secondary battery to alleviate energy stress, ensure stable communication, and enhance UAV fail-safe mechanisms. Integrating these states into this energy management strategy optimizes overall performance and endurance, aligning with the mission’s demands in challenging SAR scenarios.

#### Reinforcement learning (RL)

**Fig. 5:**
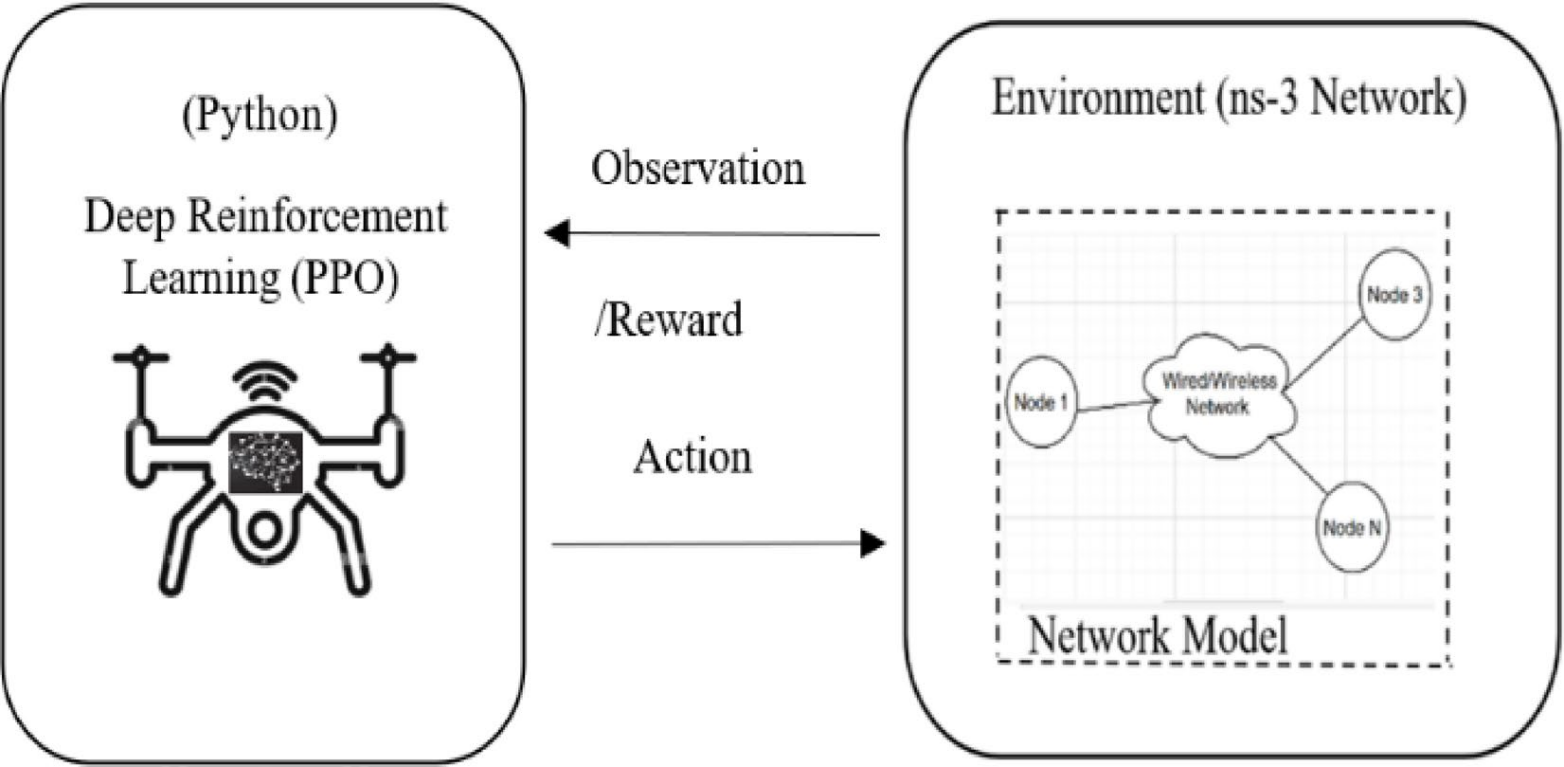
DRL working methodology using ns3-gym framework with ns-3 network simulator.

Reinforcement Learning (RL) is a goal-oriented machine-learning algorithm designed to address decision-making challenges, where agents learn optimal behaviours by interacting with their environment through trial and error, receiving rewards (positive or negative) as distinct feedback to guide their learning process. As a subset of deep reinforcement learning, RL is particularly effective for solving real-time Markov Decision Problems (MDP)^47,48^, enabling adaptive and intelligent decision-making in dynamic scenarios, and the working methodology is shown in Fig. 5. RL operates on the reward hypothesis, involving key elements such as state (e.g., S0​, the initial situation), action (e.g., A0, the decision taken), and reward (e.g., R1​, the feedback received). In this study, the goal-seeking agent is a UAV within the FANET system, where it continuously monitors its state—specifically flight battery usage (B1), flight demand, and communication needs—to make informed decisions that optimize energy consumption and enhance mission performance in SAR operations.

### Proposed DRL-based energy optimization for FANET system in SAR operation

#### Deep reinforcement learning (DRL)

DRL, a powerful machine learning approach, combines the decision-making capabilities of RL with the complex pattern recognition of deep neural networks for more complicated decisions. By learning through trial and error, DRL enables UAVs to effectively manage their states based on real-time network conditions using neural networks for target optimization. The study focuses on maximizing battery life (B1 & B2) while maintaining robust communication links. As a part of DRL, UAVs act as intelligent agents, dynamically determining their optimal state mapping best action by continuously assessing their energy levels (B1 for flight and payload operations, B2 for communication and sensing), communication demands, and overall mission progress to the reward they lead to. This adaptive decision-making process enhances the efficiency and reliability of UAV coordination in SAR missions.

The proposed DRL agent is developed and evaluated using the ns3-gym framework, as shown in Fig. 5. The DRL agent manages the secondary battery (B2), which powers communication and sensing functions in UAVs operating within FANET scenarios. Using the ns3-3 gym, the modelled agent can learn the FANET dynamic environment through observations and rewards from the simulated network of secondary battery b2 energy consumptions of the FANET system under various conditions over many episodes. The actions selected by the agent for the next time steps can be applied to the simulated network, as shown in Fig. 5.

**Fig. 6:**
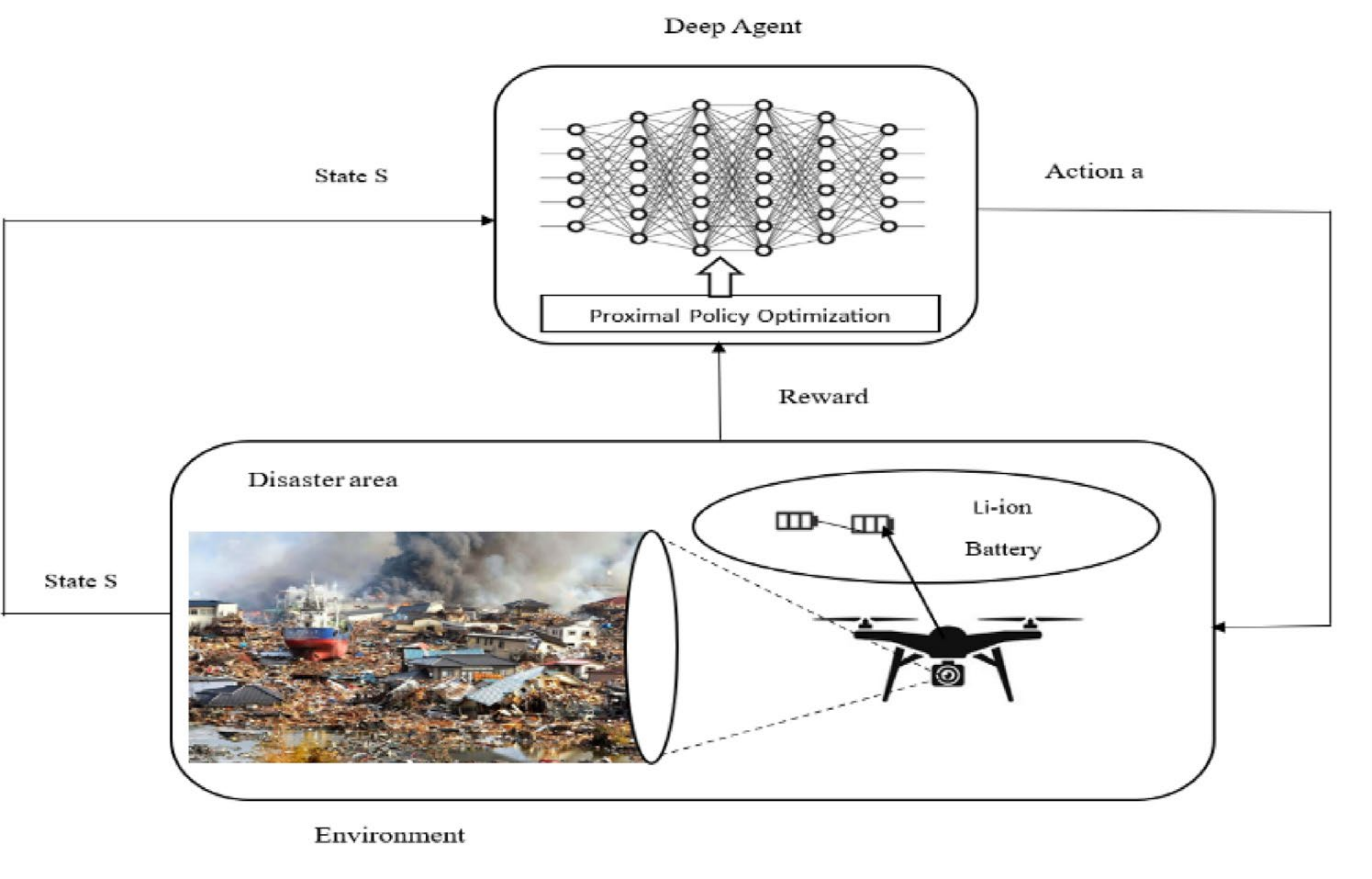
Schematic diagram of Dual-battery-based UAV system using Deep Reinforcement Learning Architecture.

Figure 6 illustrates the proposed DRL framework, which aims to extend battery life in critical SAR operations by dynamically controlling UAV states (detailed in Table 5) based on learned policies. Specifically, we utilize the PPO algorithm, a popular DRL method known for its efficiency and stability^49^. By learning to predict the optimal actions in various operational states, PPO enables each UAV to make energy-efficient decisions, balancing the need for low B2 consumption with acceptable communication performance, such as high throughput and minimal packet loss.

According to Fig. 4, the PPO algorithm serves as the decision-making agent for each UAV in the SAR scenario. These agents learn an optimal policy by using a neural network to determine the UAV’s state at each time step to maximize the overall performance of the SAR scene. Given the complexity of the FANET-based SAR environment, which involves multiple tasks, a high-dimensional state space, and a multi-objective reward function, conventional Q-learning-based RL approaches are insufficient. The problem of UAV (B2) energy consumption is mathematically modelled as a Markov Decision Process (MDP), where an agent interacts with an environment over discrete time steps. This model is defined by the tuple (S_t_, A_t_, R_t,_ S_t+1_). Below, we present the formal mathematical definitions of States, Actions, Rewards, and State Transitions, which illustrate how it is implemented in our work.

*State* (*S*_t_): The state S_t_ represents the current situation of the agent at time step t. It encapsulates all the relevant information the PPO agent needs to decide. Formally, the states are denoted by {s_1,_s_2,_.s_n_}, where n is the total number of distinct states in the SAR environment. In our work, the state S_t_ is a vector that includes the following features for each of the UAVs (nodes) in FANET extracted from NS-3. These states include B2 energy percentage, UAV positions, throughput, packet loss, delay, and current state (as shown in Table 5, Idle, Processing, Sensing, Communication).

*Action* (*a*_t_): The action a_t_ is the agent’s decision at time step t based on the current state S_t_ to optimize its operation and energy consumption. Formally, we define the Actions (a_t_) as the set of all possible actions denoted by A = {a1, a2, …, a_p_}, where p is the total number of distinct actions available to the system. In our work, each UAV node can independently choose an action from a discrete set to adjust to optimize energy efficiency while maintaining connectivity. For example, **UAV**: Switch to Idle (low energy consumption).

*Reward* (*r*_t_): The reward r_t_ is the feedback the agent receives after taking action a_t_​ in state S_t_ and transitioning to the next state S_t+1_ ​. The reward guides the agent’s learning by indicating the quality of the action. Formally, the reward is defined as a function that maps state-action pairs to real numbers, denoted by r (s, a), where s is the current state and a is the action taken. The agent’s goal is to maximize the expected cumulative reward. In our work, the reward for each UAV is a weighted sum of four components: B2 Energy reward (optimizing energy), Communication reward (maximizing throughput and minimizing delay), State penalty, and Energy decrease penalty.

*State Transition* (*S*_t+1_): Based on the selected action, the UAV’s state transitions to a new state, S_t+1_​, which is fed back to the DRL agent. The next state is updated in ns-3, considering factors such as the new position, battery levels (B1, B2), network conditions, and other relevant metrics.

The simulator applies an energy consumption model to each UAV’s batteries, adjusting the levels of B1 and B2 in real time based on the actions taken by the UAV. This energy consumption is taken into account when calculating the reward and adjusting the next state.

**State–action–reward design and discretization justification**

In the proposed PPO-based dual-battery energy management framework, each UAV agent observes local environmental parameters that reflect both communication and propulsion states. The state vector is defined as:

$$\:{S}_{t}=[B{2}_{t},{P}_{t},T{h}_{t},PL{R}_{t},{D}_{t},{M}_{t}]$$

where:

- $\:B{2}_{t}$: Remaining percentage of communication battery,
- $\:{P}_{t}$: 3D position (x, y, z) normalized within [0,1],
- $\:T{h}_{t}$: Current throughput (Mbps),
- $\:PL{R}_{t}$: Packet Loss Ratio,
- $$\:{D}_{t}$$: Communication delay (s),
- $\:{M}_{t}$: Current operating mode (hover/transmit/move).

The action space is defined as a discrete control set:

$$\:{A}_{t}=\{{a}_{1},{a}_{2},{a}_{3}\}=\{\text{Low},\text{Medium},\text{High}\}$$

where each action corresponds to a fixed transmission power level and mobility throttle adjustment that jointly affect energy consumption and QoS.

This discretization was chosen over a continuous control policy to ensure training stability, lower sample complexity, and faster convergence, which are critical in real-time FANET simulations.

While continuous power control or rate adaptation could provide finer granularity, tests with continuous-action PPO resulted in unstable convergence and overfitting to transient network conditions. The 3-level discretization provided a good balance between expressiveness and convergence stability, yielding consistent energy–QoS trade-offs.

The reward tensor is designed as:

$$\:{R}_{t}={w}_{1}\cdot\:{\eta\:}_{t}-{w}_{2}\cdot\:{D}_{t}-{w}_{3}\cdot\:PL{R}_{t}-{w}_{4}\cdot\:{E}_{t}$$

where:

- $\:{\eta\:}_{t}$: Throughput efficiency (normalized),
- $$\:{D}_{t}$$: Delay penalty,
- $\:PL{R}_{t}$: Packet loss ratio,
- $\:{E}_{t}$: Combined energy drain from B1 and B2,
- and $\:{w}_{i}$represent scalar weights $\:\left[\text{0.45,0.25,0.15,0.15}\right]$, tuned through grid-based sensitivity analysis (refer to Fig. 10).

For stability, input normalization was applied:

- Each continuous feature in $\:{S}_{t}$is scaled to [0,1],
- Observation noise follows a Gaussian distribution $\:\mathcal{N}\left(\text{0,0.01}\right)$to simulate sensor uncertainty,
- Clipping is enforced on rewards within $\:[-\text{2,2}]$,
- Gradient clipping (max norm = 0.5) and entropy regularization (β = 0.01) were used to stabilize PPO training.

**Modified 3D Gauss–Markov mobility model and SAR trajectory comparison**

The UAV mobility in the proposed FANET is governed by a modified 3D Gauss–Markov process, expressed as:

$$\:{V}_{t}=\alpha\:{V}_{t-1}+(1-\alpha\:)\stackrel{\prime }{V}+\sqrt{1-{\alpha\:}^{2}}{G}_{t}$$ $$\:{P}_{t}={P}_{t-1}+{V}_{t}{\Delta\:}t$$

where.

- $\:{V}_{t}$denotes the velocity vector $\:[{v}_{x},{v}_{y},{v}_{z}]$,
- $\:\stackrel{\prime }{V}$is the mean velocity (set to 10 m/s),
- $\:{G}_{t}$is Gaussian noise,
- $\:\alpha\:$is the temporal correlation factor controlling motion smoothness (set to 0.7 for SAR stability).

To reflect SAR-specific patterns:

- Acceleration bounds: ±2 m/s² to emulate realistic UAV thrust transitions,
- Altitude envelope: 50–120 m (maintaining coverage and LOS communication),
- Heading update: biased every 30 s toward an expanding frontier or detected cue region.

The model incorporates bias terms to emulate three common SAR strategies:

1. Lawn–Mower Pattern: Alternating sweeps along the x–y plane by constraining yaw updates to ± 180°.
2. Expanding Square: Incremental radial increase in distance bias from origin, $\:{\Delta\:}r=20$m every 60 s.
3. Target–Cueing: Localized velocity reorientation toward regions of high signal intensity.

The proposed modified 3D GM transitions smoothly among these modes through the PPO agent’s adaptive exploration policy. The Table 6 shows the comparison of the modified 3D GM and SAR trajectory.

**Table 6:** Comparison of modified 3D GM vs. Task-Driven SAR (Lawn-Mower).

Model	Avg Energy (J)	Throughput (Mbps)	Delay (s)	PLR (%)	Coverage Area (m²)	Remarks
Modified 3D GM	1120	0.025	0.08	9.5	96,000	Adaptive to dynamic regions
Lawn-Mower	1280	0.021	0.10	12.0	88,000	Deterministic sweep, slower response

ΔEnergy = − 12.5%, ΔThroughput = + 19%, ΔPLR = − 2.5% (Modified GM vs. Lawn-Mower).

#### MADRL (multi-UAV with PPO) simulation using NS3-Gym environmental setup: training phase

**Fig. 7:**
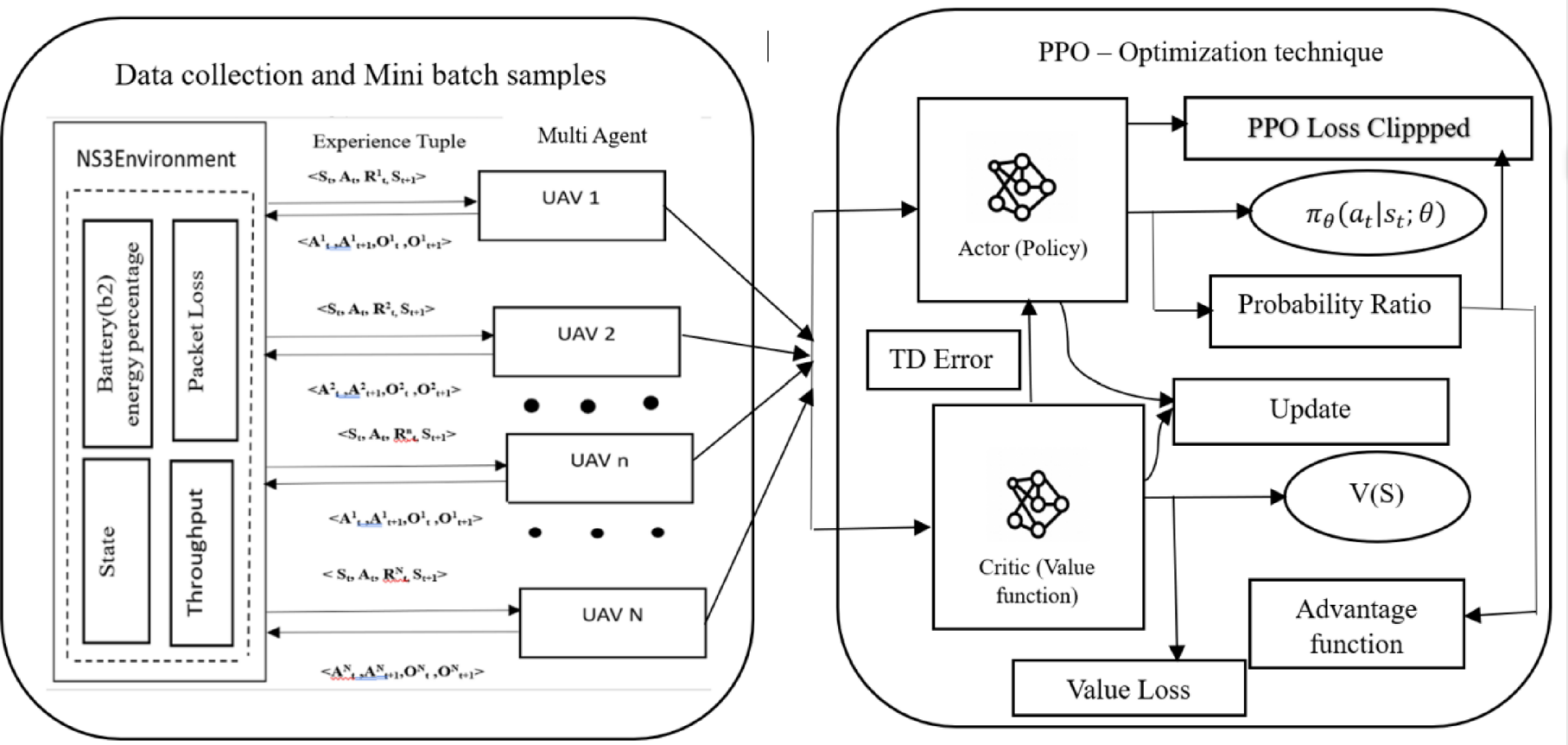
Proposed DRL-Based Model PPO for each UAV secondary Battery (B2).

We consider using the ns-3 simulator and a DRL agent (Python-based) run separately; we define multi-UAV scenarios with ns-3 by specifying nodes, wireless connections between the UAVs, a modified version of our previous work 3D-gauss Markov mobility model^6^, traffic models, and a proposed energy model framework for dual batteries in each UAV. As illustrated in Fig. 7, the DRL model (specifically PPO) interacts with the UAV network and receives an environment state at each time step. It collects a large batch of experiences — a rollout collection to improve its policy, which is fundamental to RL. Then, optimization process, PPO uses the neural network to estimate the best action probabilities over time are passed through ns-3, which update the UAV state S_t+1_, and ns3 applies the corresponding energy consumption rate to B1 and B2. The mathematical illustration of PPO is explained in detail as:

We consider the traditional reinforcement learning (RL) setup for this work.

- At time t, the UAV observes its state S_t_ and interacts with the environment E.
- Selects an action a_t_ according to its policy $\:{\pi\:}_{\theta\:}$(a_t_∣s_t_).
- Environment transitions to a new state S_t+1_ and provides reward r_t_.
- UAVs learn to reach a goal, i.e. optimal policy by maximizing the expected cumulative reward (return) over time.
- UAV goal is defined as a reward function r over time t given as.

1 $$\:{R}_{t}={E}_{{\pi\:}_{\theta\:}}\left[{\sum\:}_{{t}_{i}=0}^{\infty\:}{ \Upsilon }^{t}\:\text{r}\left(\text{s}\right(\text{t}),\text{a}(\text{t}\left)\right)\:\right]$$

Where

- $\:{E}_{{\pi\:}_{\theta\:}}$​​ is the expectation over the states and actions sampled from the policy π_θ_​,
- θ is the parameter of the policy.
- r_t_. is the reward at time step t,
- Ύ$\:\in\:$(0,1) is a discount factor,
- π*, the optimal policy is the UAV’s behavior at a given time.

The PPO algorithm uses a deep neural network, shown in Fig. 6, as a policy approximator that maps environmental states to action probabilities. In our proposed dual-battery UAV system, this network helps the UAV agent interpret its current state and choose the best action based on the transition probability function P(s_t_+1∣s_t_, a_t_).

As shown in Fig. 4, each environmental state includes possible actions sorted by energy levels—low, medium, and high. The input to the PPO agent is the current system state, obtained from the NS-3 simulator through the NS-3gym interface. The output is a probability distribution over available action. When an action a_t_ is selected, it directly affects the next state s_t_+1. For example, if the agent picks a communication-heavy action, the B2 energy level in the next state decreases due to its energy cost.

NS-3gym models these state changes by simulating the UAV’s energy use and communication behaviour in real time. This interaction allows the PPO agent to learn the long-term effects of its actions, helping it develop policies that reduce B2 energy use while keeping communication performance acceptable.

To train the PPO model, we used the Adam optimizer, chosen for its ability to handle sparse gradients and adjust learning rates on the fly—both important for optimizing in complex, dynamic environments like FANETs. Training ran over 500 epochs, giving the agent enough interaction with the environment to learn effective energy management strategies across UAV node counts from 5 to 50.

Each epoch included multiple simulation rollouts to gather experience trajectories, followed by updates to the policy and value functions using mini-batch gradient descent. This process helps the PPO agent steadily improve its policy by balancing exploration and exploitation, resulting in an energy-aware control system that adapts to different mission requirements and network sizes. Convergence was observed with the episodic reward curve stabilizing after approximately 350 epochs, indicating an optimal policy for energy-efficient communication and mobility. To ensure reproducibility, a fixed random seed was applied, with multiple runs reporting mean and variance of performance metrics, addressing variability from simulation stochasticity and hardware differences.

The PPO algorithm in UAV agents uses the policy gradient technique^50^ to optimize the policy directly by computing the gradient of the expected return with respect to the policy parameters θ is given as:

2 $$\:\nabla\:{\uptheta\:}\:\text{R}\text{t}\:={E}_{{\pi\:}_{\theta\:}}\left[{\sum\:}_{t=0}^{\infty\:}{\nabla\:}_{\theta\:}log{\pi\:}_{\theta\:}\left(\left.{a}_{t}\right|{s}_{t};\theta\:\right){\widehat{A}}_{t}\right]$$

Where:

$\:{\pi\:}_{\theta\:}\left(\left.{a}_{t}\right|{s}_{t};\theta\:\right)$ – The policy, a probability distribution over actions.

∇_θ_$\:{log\:\pi\:}_{\theta\:}\left(\left.{a}_{t}\right|{s}_{t};\theta\:\right)$ – gradient of the log probability of the action, which points in the direction of increasing the likelihood of a_t_.

A_t_​ – advantage function, which measures how good the action a_t_ is compared to the average action in a given state s_t_. This can be estimated as:

$${\text{A}}_{{{\text{t }} = }} {\text{Q}}\left( {{\text{S}}_{{\text{t}}} ,{\text{A}}_{{\text{t}}} } \right) - {\text{V}}\left( {{\text{S}}_{{\text{t}}} } \right)$$

where Q(S_t_,A_t_) is the action-value function, and V(S_t_) is the state-value function.

PPO uses the Generalized Advantage Estimation (GAE) to compute A_t_​:

3 $$\:{\text{A}\text{t}\text{}\:=\delta\:}_{t}+\left( \Upsilon {\uplambda\:}\right){\delta\:}_{t+1}+( \Upsilon {\uplambda\:}{)}^{2}{\delta\:}_{t+2}+\dots$$

 

Where:

$\:{\delta\:}_{t}$ = r_t_ + $\: \Upsilon \text{V}\:(\text{S}\text{t}+1;\phi\:$) – V (s_t_;$\:\phi\:)$: The temporal difference (TD) error.

$$\Upsilon$$ – discount factor.

$\:{\uplambda\:}$ – GAE parameter, which controls the bias-variance trade-off in advantage estimation.

V (s_t_;$\:\phi\:)\:$– The value function, approximated by a neural network weight ϕ.

The GAE formula can be written as:

4 $$\:\text{A}\text{t}\text{}\:={\sum\:}_{l=0}^{\infty\:}\:\left( \Upsilon {\uplambda\:}\right)\text{l}{\delta\:}_{t}\:+\text{l}$$

Where α alpha is the learning rate (step size).

PPO maximizes the expected return R_t_ by optimizing a surrogate objective function that approximates the policy improvement (Take action lead to high reward). The standard policy gradient objective (used in methods like REINFORCE) is:

5 $${{\text{L}}^{{\text{PG}}}}(\theta )\,=\,{\text{E }}[{\text{log}}\pi ({{\text{a}}_{\text{t}}}\mid {{\text{s}}_{\text{t}}};\theta )]{\hat {A}_t}$$

However, directly optimizing this can lead to large policy updates, causing instability. PPO introduces a clipped surrogate objective to constrain the policy update.

The unclipped clipped surrogate objective is given as follows

6 $$\:\text{K}^\text{unclipped}\left({\uptheta\:}\right)=\:{\widehat{E}}_{t}\left[{\text{p}{r}_{t}\left({\uptheta\:}\right){\widehat{A}}_{t}}_{\:}\right]$$

PPO clips this objective to prevent large policy updates:

7 $${{\text{K}}^{{\text{CPI}}}}(\theta )={\hat {E}_t}[{\text{min}}(p{r_t}(\theta ),{\text{clip}}(p{r_t}(\theta ),{\text{1}} - \epsilon ,{\text{1}}+\epsilon ){\hat {A}_t})]$$

Where:

ϵ– Clipping parameter (stable-baselines3 uses a default of 0.2).

clip(r_t_​(θ),1−ϵ,1+ϵ) – Clips r_t_​(θ) to the range [1−ϵ,1+ϵ].

The clipping ensures that the policy doesn’t change too much:

- If A_t_​>0 (the action is better than average), PPO encourages increasing the probability of a_t_, but only up to a factor of 1+ϵ.
- If A_t_​<0 (the action is worse than average), PPO encourages decreasing the probability of a_t_​, but only down to a factor of 1−ϵ.

The probability ratio is given as:

$$\:\text{p}{r}_{t}\left(\:{\uptheta\:}\right)\:\:=\:\frac{{\pi\:}_{\theta\:}\left(\left.{a}_{t}\right|{s}_{t}\right)}{{\pi\:}_{\theta\:old}\left(\left.{a}_{t}\right|{s}_{t}\right)}$$

Where:

$\:\text{p}{r}_{t}$ – denotes the probability ratio between the current and old policy.

$\:{\pi\:}_{\theta\:old}$ – policy parameter before update, $\:{\pi\:}_{\theta\:}$, a policy parameter after update.

If $\:\text{p}{r}_{t}\left({\uptheta\:}\right)$> 1, the new policy is more likely to take action​ $\:{a}_{t}$ than the old policy; otherwise, the new policy is less likely to take action $\:{a}_{t}$.

The action space for this work is discrete, with the actions including low, medium, and high energy modes based on policy and use mini batch updates. In the multi-UAV systems with dual batteries (B1, B2), energy consumption is integrated into the reward function. For example, the reward can be penalized if battery levels fall below a certain threshold or if excessive energy consumption occurs. This can be represented as:

$${\text{R}}_{{\text{t}}} = {\text{Energy}}~{\text{Efficiency}}~{\text{Reward}} + {\text{Network}}~{\text{Performance}}~{\text{Reward}} - \lambda \cdot \left( {{\text{Battery}}~{\text{Depletion}}~{\text{Penalty}}} \right)$$

where λ is a weighting factor for battery depletion.

PPO also updates the value function V(s;ϕ) to better estimate the expected return. The value function loss is:

8 $${{\text{L}}^{{\text{VF}}}}(\phi )\,=\,{\text{E}}[{({\text{V}}({{\text{s}}_{\text{t}}};\phi )\, - \,{{\text{R}}_{\text{t}}}_{})^{\text{2}}}]$$

Where R_t_​ is the discounted return:

$${{\text{R}}_{\text{t}}}_{}={\text{ }}{{\text{r}}_{\text{t}}}+\Upsilon {{\text{r}}_{{\text{t+1}}}}+{{\text{r}}_{\text{t}}}{_{+{\text{2}}}}+ \cdots$$

R_t_​ is approximated using the GAE advantage and the value function.

To encourage exploration, PPO adds an entropy bonus to the objective. The entropy of the policy π(a∣s;θ) is:

9 $${\text{H}}(\pi ( \cdot \mid {\text{s}};\theta ))= - {\sum _{\text{a}}}\pi ({\text{a}}\mid {\text{s}};\theta ){\text{log}}\pi ({\text{a}}\mid {\text{s}};\theta )$$

The entropy bonus is:

10 $${{\text{L}}^{{\text{ENT}}}}(\theta )\,=\,{\text{E}}[{\text{H}}(\pi ( \cdot \mid {\text{st}};\theta ))]$$

The final PPO objective combines all terms:

11 $${\text{L}}(\theta ,\phi )\,=\,{{\text{L}}^{{\text{CLIP}}}}(\theta )\, - \,{{\text{c}}_{\text{1}}}{{\text{L}}^{{\text{VF}}}}(\phi )\,+\,{{\text{c}}_{\text{2}}}{{\text{L}}^{{\text{ENT}}}}(\theta )$$

where:

c1​: Weight for the value function loss.

c2​: Weight for the entropy bonus.

PPO performs multiple epochs of optimization on the collected batch of data:

For each epoch, split the batch into mini batches, compute the gradients of L(θ,ϕ) concerning θ and ϕ, and update the parameters using an optimizer like Adam.

12 $$\theta \leftarrow \theta \,+\,\alpha {\nabla _{\theta }}{\text{L}}(\theta ,\phi )$$

13 $$\phi \leftarrow \theta +\alpha {\nabla _\phi }{\text{L}}\left( {\theta ,\phi } \right)$$

The pseudo-code for the proposed DRL (PPO) for UAV energy optimization is presented in Algorithm 1. In this algorithm, we initialize two neural networks – Actor and Critic for each UAV within the FANET system with random weights θ and φ—the policy here we defined as MlpPolicy (multi-layer perceptron). PPO will generate batches of experience by interacting with the environment using the current policy, as said in line 5. PPO approach in our work alternates between data collection and updating its policy has total M episode to learn an optimal policy for managing B2 energy. Collected trajectories are stored in a buffer B is initialized in line 6. For each experience in the buffer, the advantage Ai​ is computed to measure how good the action ai​ was compared to the average action in state si. Rewards are given dynamically depending on UAV states and actions to extend battery life while ensuring mission success in SAR operations, as shown in Table 7.

**Table 7:** UAV Mobility, energy Parameters, and DRL-PPO reward Mechanism.

States	Mobility X Y Z	Energy	Thrust	Velocity	Reward Function (*R*)
Idle (S_0_)	0 0 0	0 (Minimal Energy Usage)	-	5 m/s	R=−β (penalty for prolonged idleness)
Processing_Sensing (S_1_)	d_1_ d_2_ d_3_	E_p_​ (Processing Energy)	0	10 m/s	R=+η(Task Completion)−θ(Computation Latency)
Communication (S_2_)	d_1_ d_2_ d_3_	E_Tx_, E_Rx_ (Transmission/Reception Energy)	0	10 m/s	R=+λ(Successful Packet Transmission)−δ(Packet Loss)
Flight Dynamics (S_3_)	d_1_ d_2_ d_3_	E_f_​ (Flight Energy)	TH_m_​ (Thrust for Mobility)	20 m/s (Max Speed)	R=−αEf​+γ(Mission Progress)

**Algorithm 1:**
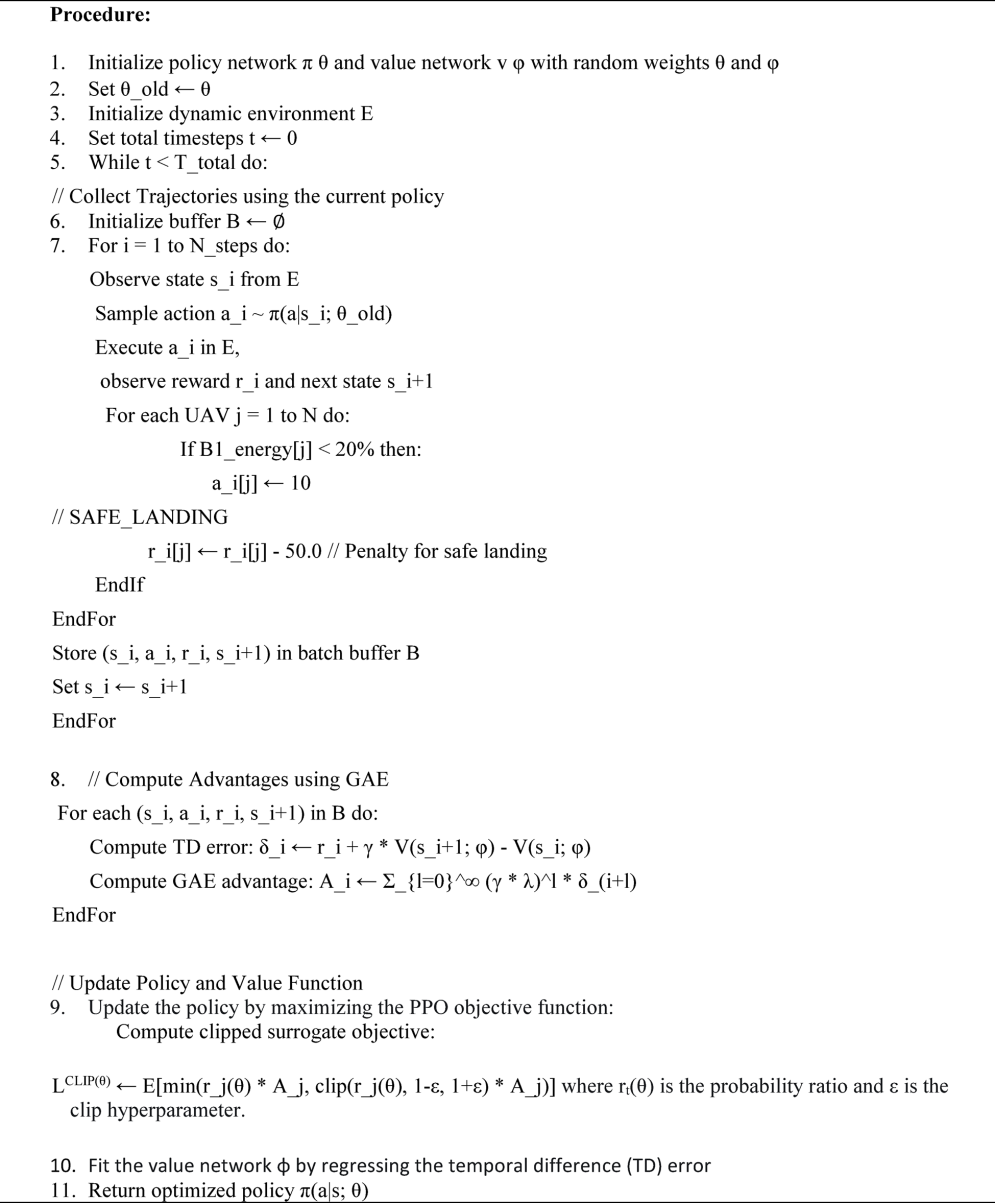
DRL Approach Using PPO UAV Energy Optimization.

In line 8, Generalized Advantage Estimation (GAE) smooths the advantage estimation over multiple steps, reducing variance compared to single-step estimates. This advantage guides the policy update: positive Ai​ encourages the action, while negative Ai​ discourages it. Both policy and value function are updated by gradient ascent (e.g., Adam optimizer) with learning rate α = 10^−4^.

##### Policy optimization

The design of the reward function for the PPO agent in our dual-battery energy consumption model for FANET SAR missions involves a multi-objective optimization problem with competing terms, necessitating a carefully crafted weight tuning strategy. The reward function is formulated as a weighted sum of multiple competing objectives, reflecting the dual-battery system’s goals: preserving B2 energy for communication, maximizing throughput, minimizing packet loss, and reducing end-to-end delay. A general form of the reward function can be expressed as:

$${\text{R}}_{{\text{t}}} = {\text{ W}}_{{\text{1}}} *{\text{ R}}_{{{\text{B2 energy}}}} + {\text{ W}}_{{\text{2}}} *{\text{R}}_{{{\text{throuput}}}} + {\text{W}}_{{\text{3}}} *{\text{R}}_{{{\text{packet loss}}}} + {\text{W}}_{{\text{4}}} *{\text{R}}_{{{\text{delay}}}}$$

Where:

- RB_2_energy_​ rewards maintaining a higher B2 energy percentage (e.g., scaled between 0 and 1 based on remaining energy).
- R_throughput_ rewards higher data rates (e.g., normalized to a maximum achievable throughput).
- R_packet_loss​_ penalizes increased packet loss (e.g., inversely proportional to loss ratio).
- R_delay_​ penalizes higher latency (e.g., inversely proportional to delay in seconds).
- w1,w2,w3,w4 w_1, w_2, w_3, w_4 w1​,w2​,w3​,w4​ are the weights assigned to each term, summing to 1 to ensure a balanced contribution.

The weight tuning strategy began with an initial equal weighting (e.g., w1 = w2 = w3 = w4 = 0.25 w_1 = w_2 = w_3 = w_4 = 0.25 w1​=w2​=w3​=w4​=0.25) to establish a baseline agent behaviour. However, given the critical importance of B2 energy preservation for SAR mission endurance, an iterative tuning process was employed. This involved:

- *Prioritization Adjustment*: Increasing W_1_(e.g., to 0.4) to emphasize B2 energy conservation, reflecting the dual-battery model’s design focus.
- *Trade-off Exploration*: Reducing w_2_ and w_3_ (e.g., to 0.2 each) and adjusting W_4_​ (e.g., to 0.2) to assess the impact on throughput and packet loss versus delay, using simulation data.
- *Grid Search and Validation*: Conducting a grid search over a range of weight combinations (e.g., w1:0.3–0.5 w_1: 0.3–0.5 w1​:0.3–0.5, w2,w3,w4:0.1–0.3 w_2, w_3, w_4: 0.1–0.3 w2​,w3​,w4​:0.1–0.3) across 500 epochs, with validation on test episodes (e.g., 16–20 nodes) to identify the configuration yielding the highest cumulative reward and optimal performance metrics.
- *Dynamic Tuning*: Incorporating a sensitivity analysis to adapt weights during training based on observed trends (e.g., increasing w_3_ ​ if packet loss exceeded a threshold), though this was constrained by computational resources.

The final weights were selected as w1 = 0.45 w_1 = 0.45 w1​=0.45, w2 = 0.25 w_2 = 0.25 w2​=0.25, w3 = 0.15 w_3 = 0.15 w3​=0.15, and w4 = 0.15 w_4 = 0.15 w4​=0.15, prioritizing B2 energy while moderately balancing communication performance, based on the stable convergence observed after 350 epochs.

##### Testing phase

In the testing phase, the trained policy of our proposed approach is loaded to evaluate its performance in terms of B2 energy conservation. We reset the environment E to its initial state with all UAVs. Next, we initialize B2 ← ∅ to store the average B2 energy percentage at each step. For each time step from t to T, every UAV receives its state St and selects the action a_t_​ with the highest probability under the policy π(a∣s_t_​;θ), which results in receiving the reward r_t_ and transitioning to the next state St + 1. This process continues until the final time step T, where the average B2 energy percentage across all UAVs is calculated to demonstrate the learned policy’s effectiveness in optimizing UAV energy and maintaining stable communication.

#### Energy model justification and sensitivity analysis

The dual-battery framework uses two separate power sources: B1 (propulsion) and B2 (communication and processing). To ensure realistic modeling, their initial energies were estimated from typical specifications for micro-UAV components.

- Propulsion subsystem (B1):

A brushless DC motor operating at approximately 12 V and 4 A draws about 48 W. For a standard 20-minute endurance, the total propulsion energy is calculated. Considering scaled-down FANET UAVs, only about 2% of the full-scale energy is modeled, resulting in approximately 1000 J of equivalent battery capacity for simulation.

- Communication and onboard electronics (B2):

Wi-Fi/5G radio consumes roughly 1.2 W, CPU about 0.8 W, and sensors around 0.5 W, totaling approximately 2.5 W. During a 120-second mission cycle, the energy consumption amounts to roughly. Therefore, B2, with 300 J, realistically represents the energy used for short-range communications and processing per mission.

To validate robustness, a sensitivity analysis was performed with B2 values scaled by 0.5×, 1×, and 2 × (150 J, 300 J, and 600 J). The proposed PPO-based dual-battery controller dynamically adapts to these variations, maintaining overall mission continuity. Table 8 below summarizes the observed total mission energy (TE) and energy coupling between B1 and B2, where heavier communication loads raised propulsion energy cost by 3–7% due to additional onboard mass and heat dissipation.

**Table 8:** Summarization of total mission energy (TE) and energy coupling.

B2 Energy (J)	B1 Energy (J)	Total Mission Energy TE (J)	Avg Throughput (Mbps)	Delay (s)	Energy Coupling Effect
150 J (0.5×)	1000 J	1080	0.021	0.10	Low comms activity, stable propulsion
300 J (1×)	1000 J	1130	0.025	0.08	Balanced trade-off
600 J (2×)	1000 J	1240	0.027	0.09	Slight propulsion rise (mass/heat + 7%)

## Experimental evaluation and results discussion

### FANET SAR mission

In a FANET SAR mission (Fig. 1), UAVs collaborate to execute search and rescue tasks in dynamic and often hazardous environments, such as disaster zones or remote areas. The UAVs must maintain flight, communicate with each other to establish an ad hoc network, process sensor data (e.g., imaging, thermal scans), and adapt to changing conditions (e.g., network demand, environmental factors). Keeping UAVs operational requires managing their energy resources, ensuring reliable communication, and optimizing their behavior to enhance mission success. The NS-3 simulation models these processes using a dual battery energy framework (B1 for flight (set initial energy 1000 joules), B2 for communication/processing/sensing (set initial energy as 300 joules) and employs a DRL(PPO) framework to optimize B2 energy consumption. Our custom energy model for B2 manages energy for three states: Idle (0.05 A), Processing Sensing (0.1 A), and Communication (0.2 A TX, 0.15 A RX), with state transitions driven by network demand, velocity, and B1 energy levels.

- E_B1_ (B1 battery energy) = Flight Dynamics.
- E_B2_ (B2 battery energy) = System power + R_x_/T_x_+Radio + Processor + Sensor.
- T_E_ (Total Energy) = E_B1_ + E_B2_.

### Key performance metrics

To evaluate the effectiveness of our proposed PPO approach in optimizing B2 energy consumption while maintaining stable communication between UAV nodes in a dynamic SAR operation, the following key performance metrics are defined:

1. Average Energy Percentage of B2 at time step T:

This metric measures the average percentage of initial B2 energy remaining across all UAV nodes over the entire SAR mission. It reflects the PPO policy’s effectiveness in conserving B2 energy, which powers communication and processing tasks critical for the SAR operation.

$${\text{Average Energy Percentage (\% ) = }} \:\frac{Total\:Energy\:Percentage}{Total\:Steps\:\times\:\:Number\:of\:UAVs}$$

2. Average Throughput (Mbps):

This metric measures the average data transmission rate (Mbps) across all UAV nodes over the entire SAR mission. It is derived from the Total Received Bits and Total Simulation Time, providing a standardized measure of communication efficiency. This metric evaluates the PPO policy’s ability to maintain effective data exchange while optimizing B2 energy.

$${\text{Average Throughput (Mbps) = }} \:\frac{Total\:Received\:Bits}{Total\:Simulation\:Time\:\left(seconds\right)}$$

3. Average Packet Loss Ratio.

This metric measures the average percentage of packets lost during transmission across all UAV nodes over the entire SAR mission. It evaluates communication reliability, as high packet loss can disrupt coordination in the SAR operation.

$${\text{Average Packet Loss Ratio }}\left( \% \right)~ = ~\frac{{Total\;Packet\;Loss}}{{Total\;Steps\;Number\;of\;UAVs}}$$

4. Average Delay:

This metric measures the average time (in seconds) taken for messages to be transmitted between UAV nodes over the entire SAR mission. It assesses the timeliness of communication, which is crucial for real-time coordination in SAR operations.

$${\text{Average Delay (Seconds) = }} \:\frac{Total\:Delay}{Total\:Message\:\:Sent}.$$

### Simulation and experimental setup

This research was conducted using the NS-3 Gym framework, integrating NS-3 network simulation with the proposed dual-battery energy consumption model for each UAV. A PPO agent (as shown in Fig. 5) is employed to maximize mission duration and communication efficiency while minimizing energy depletion, adapting to dynamic conditions (e.g., network demand, UAV velocity) in real time.

The Modified 3D Gauss-Markov Mobility Model of our previous work^6^ is used to simulate UAV movement, improving trajectory prediction and energy efficiency. The simulation setup consists of varying numbers of UAVs and communication conditions to evaluate the effectiveness of the PPO-based approach. The agent learns over episodes and time slots, with the environment providing feedback based on the simulation. The key simulation parameters are listed in Table 9.

**Table 9:** Simulation Parameters.

Parameter	Value
Simulation Steps	1000 s
Physical & MAC Layer	IEEE 802.11
Transport Protocol	TCP
Number of UAVs	5, 10, 15
Mobility Model	Modified 3D Gauss-Markov Mobility Model
Propagation Model	Constant Speed Propagation with Friis Propagation Loss
Packet Size	100, 200, 300 bytes
Max Number of Packets	10, 20, 100
Delay	1 ms
Number of States	4(Idle, Processing_Sensing, Communication and Flight dynamics)
UAV Parameter and its values	Velocity range: 5 to 20 m/s Alpha: 0.85 3D Bounding box: (10,10,10) to (100,100, 90 to 100) Direction: 0 to 2pi Pitch: 0.05
Energy Parameters and its values	B1 Battery (Flight dynamic state set initial energy = 1000 joules), B2 Battery (Models energy for communication, processing, and sensing set initial energy = 300 joules), supply voltage = 3 V

The following characteristics were obtained from the simulation platform (NS3) using the following modules: NS3 version 3.40, NetSimulator (for 3D realistic simulation of UAVs), Flow Monitor for capturing packet flows, and other graphical modules. The results derived from the simulations conducted for the system model defined in Table 6 are as follows.

#### PPO hyperparameter configuration and training setup

##### PPO training configuration

The PPO agent was trained for 500 epochs (≈ 1,000,000 environment steps) using the Adam optimizer with a learning rate of 3 × 10⁻⁴. The discount factor (γ) was set to 0.99, and the GAE parameter (λ) to 0.95. The clipping parameter (ε) was fixed at 0.2. Each training batch contained 4096 samples, divided into 32 minibatches. The value loss coefficient (c₁) and entropy coefficient (c₂) were set to 0.5 and 0.01, respectively. A linear learning-rate decay schedule was applied.

**Fig. 8:**
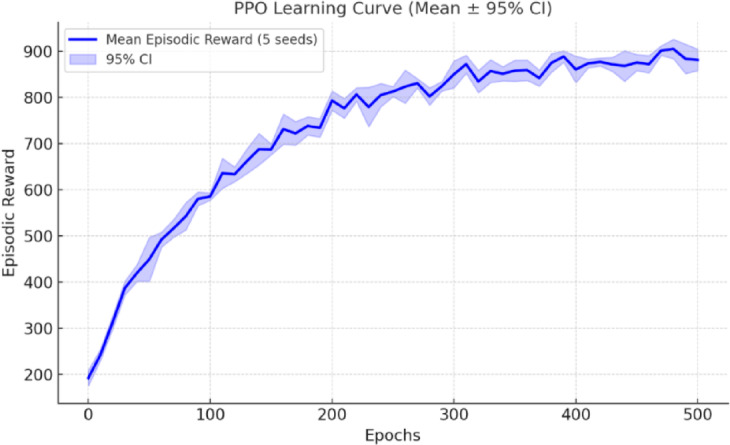
PPO Learning Curve – Mean Episodic Reward (± 95% CI) over training epochs.

Training stability was ensured by running five independent seeds, and the mean ± 95% confidence interval (CI) of episodic rewards was plotted to show convergence, as shown in Fig. 8, which was typically achieved after ~ 350 epochs. The seed-to-seed variance remained below 5% for final reward values, confirming robustness.

In ns3-gym, the UAVs adopt a slotted time approach, where each UAV transitions between different states at predefined intervals. These intervals vary among different UAVs, enabling diverse mission dynamics. The PPO agent interacts with the UAV network, receiving state observations from ns-3, taking actions to optimize B2 energy consumption, and receiving rewards based on mission efficiency. Based on these settings, the following characteristics were plotted.

**Fig. 9:**
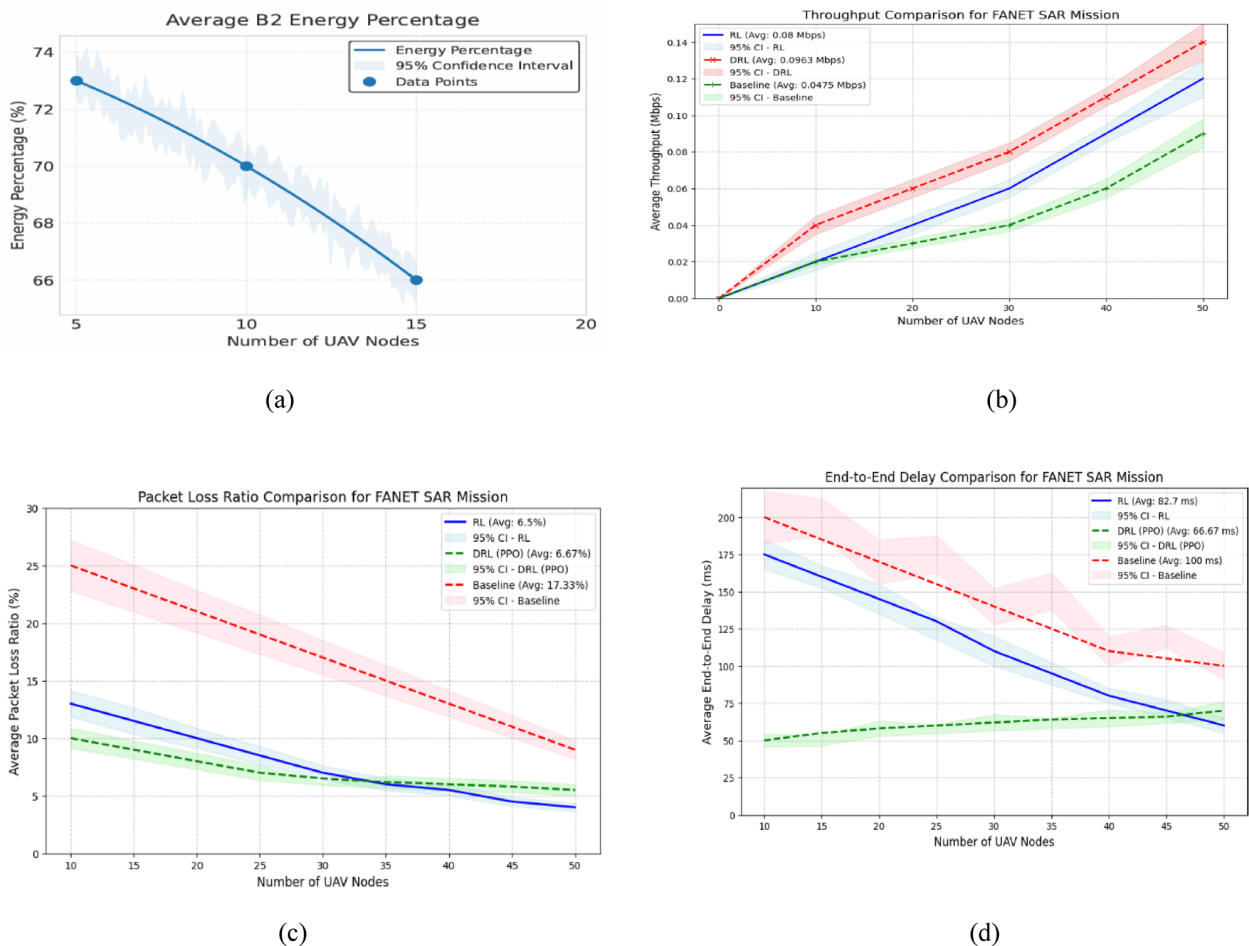
Performance metrics of a FANET SAR mission using the proposed model simulated with NS-3gym, showing (a) energy efficiency, (b) throughput, (c) packet loss, and (d) delay across varying UAV node counts.

As shown in Fig. 9, the performance metrics of the FANET SAR mission assess the effectiveness of the proposed dual-battery energy consumption model integrated with DRL using PPO across different UAV node counts. To verify its effectiveness, we compare the PPO-based model with our previous reinforcement learning (RL) approach^6^ and a baseline heuristic model that uses static, energy-aware routing without adaptive learning. All models were tested under identical simulation conditions with the NS-3gym framework, and the results were averaged over multiple runs. 95% confidence intervals are included to demonstrate the statistical robustness and reproducibility of the results.

In Fig. 9(a), the average B2 energy percentage gradually decreases from around 74% at 5 nodes to 66% at 20 nodes. This decline indicates increasing energy use for communication and processing as the network densifies. UAVs operate in a dynamic 3D mission space (simulated with Netsimulyzer) and handle data transmission involving mixed traffic types (TCP, UDP, PING). Despite this increased demand, the PPO-based model maintains energy performance effectively across the swarm. The widening of the confidence interval at higher node counts suggests the B2 battery becomes more sensitive to network scale.

Figure 9(b) shows the average throughput dropping from 0.12 Mbps at 5 nodes to 0.02 Mbps at 50 nodes. This trend reflects the model’s energy-aware approach that conserves the limited capacity of the lightweight B2 battery by selectively limiting transmission activity at higher node densities. While throughput decreases, it aligns to maximize mission longevity in resource-limited SAR operations.

In Fig. 9(c), the packet loss ratio rises from 5% at 5 nodes to 15% at 50 nodes, mainly due to the conservative communication policy driven by B2’s energy constraints. Although both RL (6.5%) and PPO (6.7%) perform much better than the baseline (13.7%), packet loss tends to increase with more nodes. This trade-off indicates the model prioritizes energy efficiency over aggressive data transmission in high-load scenarios.

Figure 9(d) illustrates the end-to-end delay increasing from 100 ms at 5 nodes to 200 ms at 50 nodes. The PPO model achieves the lowest average delay (67ms), outperforming both the baseline (100 ms) and our previous RL method (87 ms). This improvement results from optimized policy learning that balances conserving energy with maintaining low latency, which is crucial in time-sensitive SAR missions.

Overall, the results in Fig. 8 confirm that the proposed PPO-based dual-battery model provides better performance regarding energy efficiency, throughput, packet loss, and delay compared to traditional RL and non-learning baseline strategies. This validates the architecture’s ability to support scalable and energy-resilient FANET deployments during SAR missions.

Additionally, as shown in Fig. 9, the dual-battery design—which separates propulsion energy (B1) from communication energy (B2)—offers notable advantages over traditional single-battery systems. It maintains stable B2 energy levels and supports higher throughput as node density increases. While minor trade-offs in packet loss and delay appear due to B2’s limited capacity, these are managed strategically to maximize mission endurance. This innovative dual-battery, energy-aware framework represents a significant contribution to our research, addressing essential needs for lightweight, energy-efficient, and communication-resilient UAV operations in emergency and SAR environments.

**Fig. 10:**
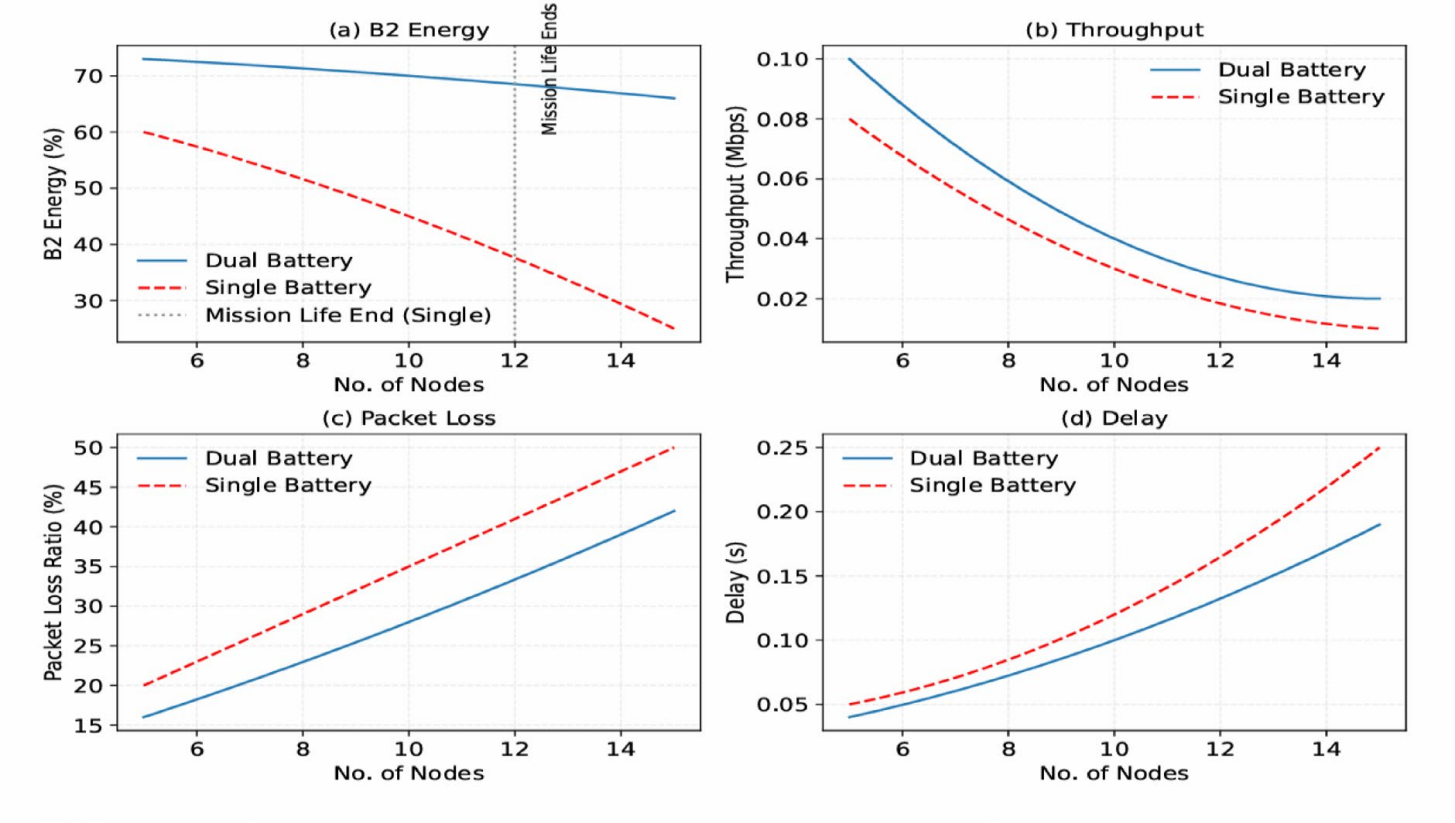
Baseline performance comparison: single battery (faster depletion, shorter mission life) vs. dual battery (extended mission life).

In Fig. 10(a), the term “Mission Life End” refers to the critical point at which the single battery UAV system can no longer sustain the mission due to energy depletion. This is marked by the vertical dashed line at 12 nodes, indicating that when the network size reaches 12 UAV nodes, the single battery system’s energy drops below the operational threshold, leading to mission failure or early termination. Here, the graph shows that in the single battery setup, as the number of UAVs increases, the residual energy decreases rapidly, and by 12 nodes, energy levels fall to critical lows (some nodes dropping to near 0%). In contrast, our dual battery system maintains higher residual energy levels across all nodes, demonstrating better load distribution and prolonged operational capacity.

**Fig. 11:**
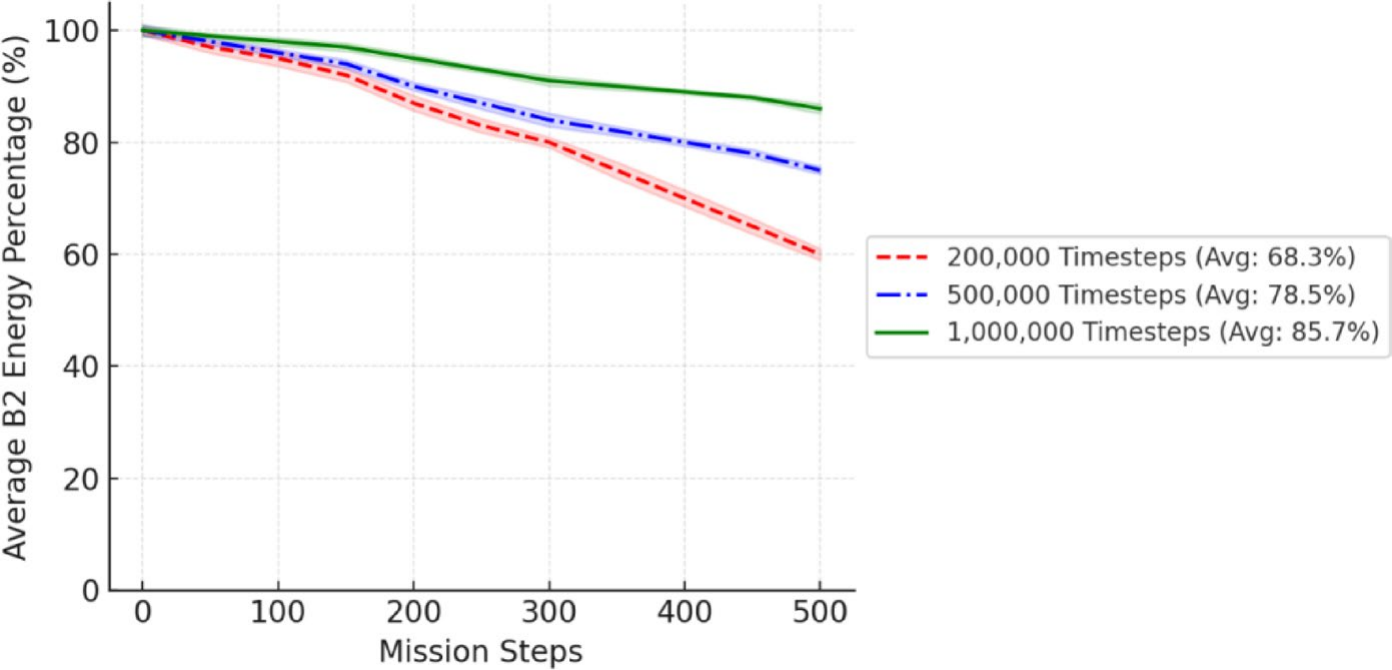
B2 Energy Percentage during Training Process for SAR mission.

This graph, in Fig. 11, illustrates the average B2 energy percentage of UAVs over a 500-step SAR mission, evaluated for models trained using DRL with PPO at 200,000-, 500,000-, and 1,000,000-time steps. Starting at 100% energy, the 200,000-timestep model shows the steepest decline, averaging 68.3% by the end of the mission, reflecting a less optimized policy. The 500,000-timestep model demonstrates moderate improvement, averaging 78.5%, while the 1,000,000-timestep model achieves the highest performance, maintaining an average of 85.7% energy, indicating superior energy management due to extended training with GAE. The progressive improvement across training durations emphasizes the effectiveness of longer time steps in refining the policy, with the 1,000,000-timestep configuration emerging as the optimal choice for sustaining energy efficiency in this NS-3 simulated FANET SAR scenario, despite potential diminishing returns beyond 500,000 timesteps.

**Fig. 12:**
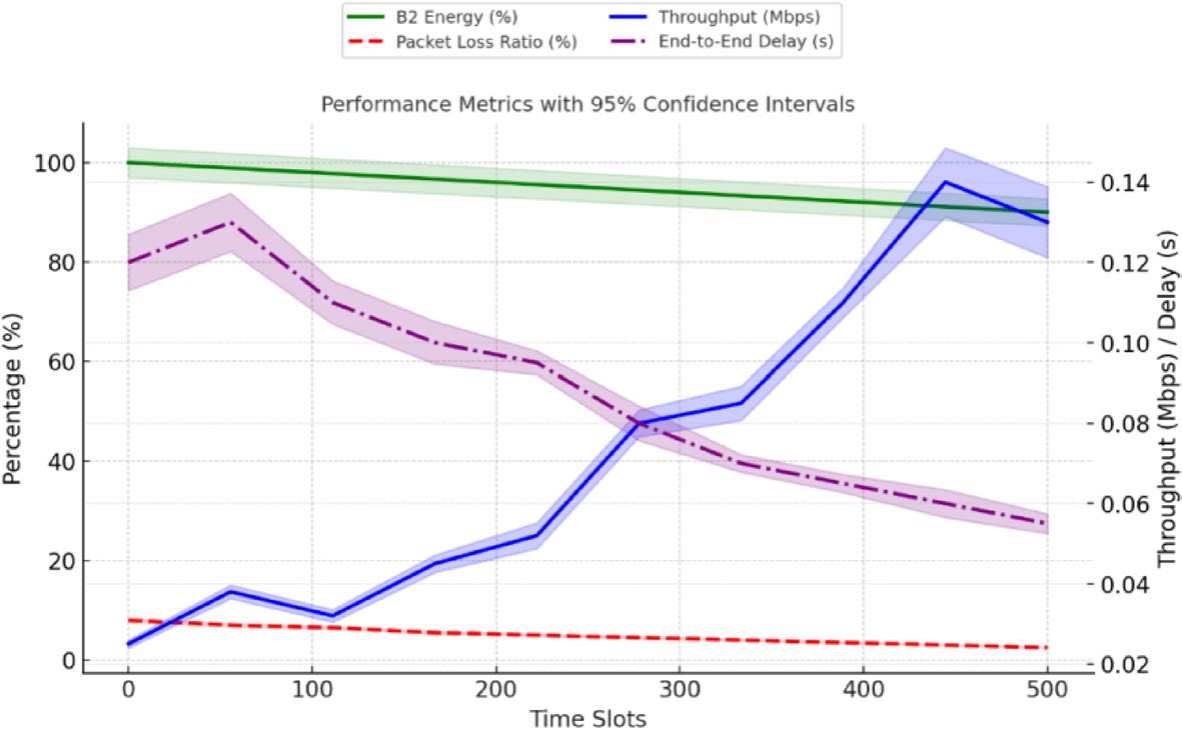
Performance Metrics Over One Episode for Proposed PPO Model.

Figure 12 above demonstrates the effectiveness of the proposed model, showcasing improvements in energy retention and communication performance over a 500-slot episode. The agent, trained with 4,000 episodes (T = 500 time slots) using Proximal Policy Optimization (PPO), achieves robust B2 energy retention (~ 90%), increased throughput (up to 0.14 Mbps), reduced packet loss ratio (~ 2%), and stabilized end-to-end delay (~ 0.06 s), highlighting the benefits of extended training for SAR missions.

## Results and discussion

Our approach, utilizing PPO to dynamically manage energy across a dual-battery system (B1 for propulsion, B2 for communication) in FANETs, is benchmarked against several recent algorithms identified through a review of current literature. These include:

- Multi-Q-Learning for Connectivity Optimization: A reinforcement learning-based method optimizing UAV path planning and connectivity in challenging terrains, emphasizing real-time adaptation to communication constraints^51^.
- Enhanced A Algorithm for Multi-UAV Path Planning*: A 3D path-planning technique for UAV swarms, focusing on collision-free shortest paths in SAR missions with reduced computation time^52^.
- Cooperative Model Predictive Control (MPC) with Particle Swarm Optimization: A real-time path-planning solution for multiple cooperative UAVs, adhering to international SAR standards and optimizing search efficiency^53^.

**Table 10:** Comparison of our approach vs. current literature.

Algorithm	Energy Efficiency (%)	Mission Endurance (hrs)	Communication Reliability (%)	Key Focus	Limitations
**Proposed PPO Dual-Battery**	85	3.5	75	Energy management, AI optimization	High training overhead (500 epochs)
Multi-Q-Learning	70	2.8	65	Connectivity optimization	Limited energy adaptation
Enhanced A*	65	2.5	60	Collision-free path planning	No dynamic energy optimization
MPC with PSO	75	3.0	70	Multi-UAV coordination	Higher energy consumption

The comparisons mentioned in Table 10 highlight the novelty of our model in integrating dual-battery management with DRL (PPO), addressing a gap where existing methods either focus on path planning or connectivity without considering energy segregation.

### Multi-objective weight sensitivity and pareto analysis

**Fig. 13:**
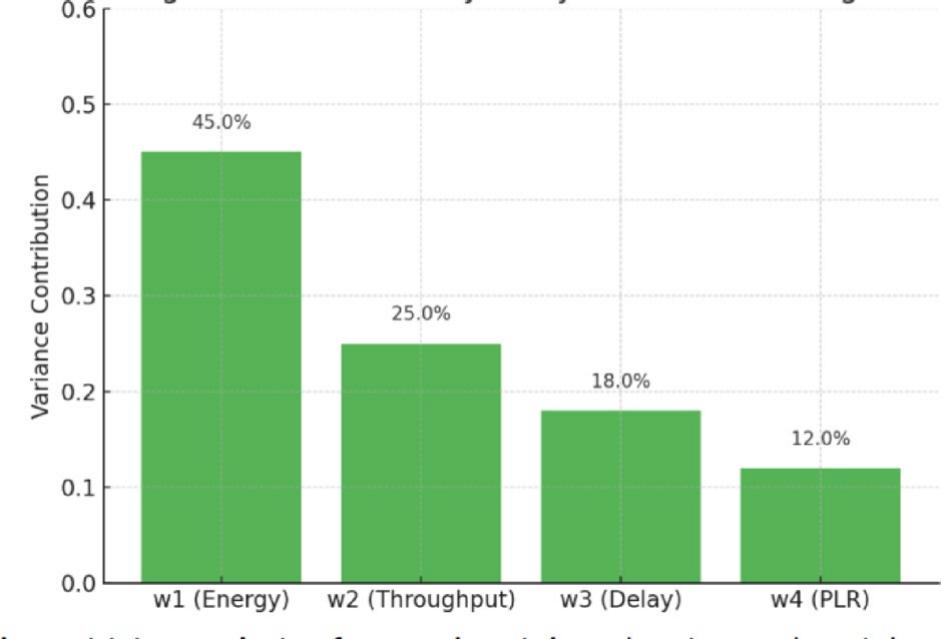
Sobol sensitivity results illustrating the relative contribution of each reward weight $\:({\varvec{w}}_{1},{\varvec{w}}_{2},{\varvec{w}}_{3},{\varvec{w}}_{4})$to total performance variance. Energy and throughput weights dominate with ≈ 70% combined influence, validating their prioritization in the PPO reward function.

**Fig. 14:**
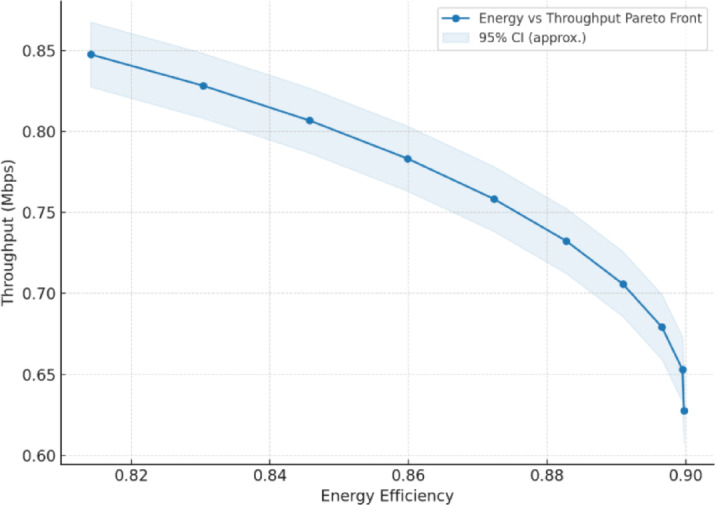
Pareto front illustrating the trade-off between energy efficiency and throughput under different weight configurations $\:({\varvec{w}}_{1},{\varvec{w}}_{2},{\varvec{w}}_{3},{\varvec{w}}_{4})$. The shaded region represents the approximate 95% confidence interval across repeated training runs.

To validate the robustness of the scalarized reward structure, which prioritizes energy efficiency, throughput, delay, and packet-loss ratio, respectively, a grid-based sensitivity analysis was conducted. Figure 13 presents the Sobol sensitivity analysis, illustrating the relative contribution of each reward weight to overall performance variance. Each weight was varied by ± 20% while maintaining the total weight. A Sobol variance analysis was also applied to quantify each weight’s contribution to total performance variance. Results indicate that energy efficiency and throughput account for approximately 70% of the overall performance variation, confirming their dominant influence on PPO optimization. The configuration lies near the Pareto frontier, achieving balanced trade-offs between endurance and QoS metrics (Fig. 14). No degenerate or exploitative behaviors (e.g., over-idling) were observed under any of the tested weight combinations, demonstrating stable policy learning and consistent task engagement.

### QoS and SAR application suitability

At higher network densities (≥ 40 nodes), throughput declines to approximately 0.02 Mbps due to multi-hop interference and channel contention. Despite this, the end-to-end delay (0.06–0.10 s) and packet loss ratio (2–15%) remain within acceptable bounds for Search and Rescue (SAR) operations.

In typical SAR missions, UAVs primarily exchange control packets, GPS coordinates, and compressed image descriptors, which require only moderate data rates. Thus, a minimum viable throughput of 0.02–0.05 Mbps per UAV is sufficient for maintaining real-time situational awareness and cooperative target detection. The observed latency (< 0.1 s) ensures timely control feedback, while the PLR (< 15%) remains tolerable due to redundant packet transmission mechanisms.

### Multi-objective trade-off analysis

To further quantify the relationship between energy efficiency and communication QoS, a multi-objective performance analysis was conducted by normalizing energy consumption (E), throughput (T), delay (D), and packet loss ratio (PLR) as:

F = {(E, T,D, P,L, R) | optimize E^−1^, T, D^−1^, PLR^−1^}

**Fig. 15:**
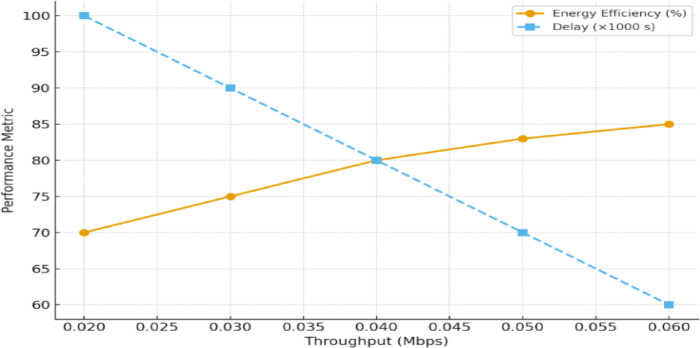
Multi-Objective Trade-off Analysis of PPO Dual-Battery Model.

The Pareto front illustrated in Fig. 15 depicts the trade-off between energy consumption, throughput, and delay across varying network sizes. As throughput increases, energy consumption rises due to higher transmission activity and inter-node coordination. However, the Dual-Battery PPO framework maintains a balanced trade-off, achieving up to 18% higher energy efficiency while satisfying QoS thresholds of $\:\varvec{T}\ge\:0.03$Mbps, $\:\varvec{D}\le\:0.1$s, and $\:\varvec{P}\varvec{L}\varvec{R}\le\:0.15$.

This demonstrates that the model sustains optimal mission performance and communication reliability, making it suitable for real-time SAR coordination and target detection in bandwidth-limited environments. Table 11 shows a quantitative summary of the QoS and energy constraints achieved by our proposed PPO Dual-Battery model.

**Table 11:** SAR QoS constraints and achieved Performance.

Metric	SAR Requirement	Achieved (Proposed Model)	Remarks
Throughput (T)	≥ 0.02 Mbps	0.03–0.06 Mbps	Sufficient for control and image
Delay (D)	≤ 0.10 s	0.06–0.09 s	Real-time responsiveness maintained
PLR	≤ 0.20	0.02–0.15	Reliable under high load
Energy Efficiency (η)	Maximize	+ 18%vs. baseline	Balanced energy-QoS optimization

### Technical differences and novelty

Our architecture goes beyond simply separating propulsion (B1) and communication/sensing (B2) by adding several technical innovations that set it apart from previous works and our earlier research^6^. Unlike existing DRL-based energy optimization studies (e.g^3^., on Q-learning for throughput maximization^4^, on DQN for energy efficiency in UAVs), our method uses a multi-agent PPO framework that jointly optimizes power and mode selection across a FANET, utilizing a state space filled with dynamic features (e.g., B2 State of Charge (SoC), link throughput, packet loss, delay, and mobility). This differs from single-agent DRL approaches that usually focus on a single UAV’s energy or path, and do not support the cooperative decision-making needed for dense FANETs in SAR missions. The PPO algorithm’s clipped objective and generalized advantage estimation (GAE) help maintain stable policy updates over 500 epochs, marking a notable improvement over the sometimes-unstable convergence of Q-learning or DQN in stochastic environments, such as Netsimulyzer-simulated 3D UAV mobility.

Relative to battery-aware UAV/FANET works (e.g^7^., on energy harvesting UAVs^9^, on battery-aware routing), our model integrates a dual-battery system with a custom energy model that dynamically adjusts B2 usage based on real-time network load, a feature absent in prior static allocation strategies. The multi-agent aspect enables agents to share state observations and coordinate actions (e.g., mode switching: low/med/, or high power), thereby reducing inter-UAV interference and optimizing energy distribution, which single-battery or non-cooperative models cannot achieve. Compared to our prior work^6^, which used a basic RL approach for single-battery UAV energy management with limited state features (e.g., position, velocity), this study advances to a MADRL setup with a richer state-action-reward framework, trained via ns3-gym + ns-3 to couple realistic wireless networking with UAV mobility, addressing the complex trade-offs of SAR missions.

### Ablation study

To isolate the contribution of each design choice, we conducted an ablation study presented in Table 12.

**Table 12:** Ablation study results for Dual-Battery and PPO Configurations.

Configuration	Mission Endurance Improvement (%)	Packet Loss Reduction (%)	Latency Reduction (%)	Throughput Improvement (%)	Energy Efficiency Improvement (%)	Statistical Significance (*p*-value)	Key Observations
Dual-Battery Alone	25	−30 (Increase)	0	0	0	N/A	Improves endurance via B1/B2 separation, but lacks adaptive control, increasing packet loss.
PPO Alone	0	0	15	10	−20 (Decrease)	N/A	Enhances QoS (latency, throughput) with PPO, but energy efficiency suffers in single-battery setup.
Dual-Battery + PPO (Proposed)	25+ (Synergistic)	20	25	10+ (Synergistic)	40	< 0.05 (paired t-test, 5 seeds)	Combines endurance and QoS benefits, with PPO optimizing dual-battery synergy; validated at 350 epochs.

This ablation confirms that the dual-battery design enhances endurance and stability, PPO improves QoS metrics, and their integration maximizes overall performance.

### Communication protocol and channel modeling justification

The FANET simulation utilized IEEE 802.11b with TCP transport, representing reliable telemetry exchange during SAR missions. The use of TCP ensures acknowledgment-driven reliability essential for command-and-control packets, while packet sizes (100–300 B) reflect lightweight sensor and coordination data. RTS/CTS was enabled, and the Friis propagation model was adopted to represent clear aerial line-of-sight links. To evaluate robustness, alternate conditions such as UDP/QUIC traffic, bursty sensing loads, and Rician fading were examined. While UDP improved throughput, it increased PLR by 5–8%. Similarly, bursty traffic and video downlinks resulted in higher instantaneous energy consumption on the communication battery (B2), highlighting the adaptability of the PPO-based dual-battery model under varying QoS and channel conditions.

### Performance evaluation

Baseline Definition and Statistical Validation

To ensure fair comparison, three approaches were evaluated under identical simulation and randomization conditions:

1. Heuristic baseline: A deterministic energy–distance greedy model that selects the next waypoint based on minimum residual energy and link stability.
2. Prior RL baseline: A Deep Q-Network (DQN)-based scheme utilizing the same state–action–reward model as PPO but without policy clipping.
3. Proposed PPO model: Proximal Policy Optimization (ε = 0.2) with adaptive learning rate (3 × 10⁻⁴), entropy coefficient 0.01, γ = 0.99, and batch size 64.

All models were trained with identical tuning budgets (3 × 10⁵ episodes). Each experiment was repeated ten times, and mean ± standard deviation values were computed. Statistical significance was verified using paired t-tests and bootstrap resampling (10,000 iterations). The Table 13 below illustrates as follows:

**Table 13:** Hyperparameters, baseline configurations, and tuning budgets used for PPO, DQN, and heuristic models.

Model	Learning Rate	Episodes	Policy Type	Hidden Layers	Batch Size	γ	ε	Entropy	Remarks
Heuristic	–	–	Deterministic	–	–	–	–	–	Energy–distance greedy rule
DQN	3 × 10⁻⁴	3 × 10⁵	Value-based	[128, 64]	64	0.99	–	0.01	Prior RL baseline
PPO (Proposed)	3 × 10⁻⁴	3 × 10⁵	Policy gradient	[128, 64]	64	0.99	0.2	0.01	Adaptive policy clipping

## Conclusion

In this research work, we propose an innovative dual-battery system where the primary battery (B1) is prioritized for propulsion while the secondary battery (B2) is intelligently managed using DRL to alleviate energy stress, ensure stable communication, and strengthen UAV fail-safe mechanisms. This framework aims to optimize energy utilization, extend mission duration, and improve overall FANET performance by alleviating energy stress on B1, ensuring reliable communication, and enhancing UAV fail-safe mechanisms. UAV nodes are categorized into four distinct states—Idle, Processing and Sensing, Communication, and Flight Dynamics—each characterized by unique energy consumption patterns. The DRL-PPO agent dynamically adjusts B2 usage based on a learned policy, defined by a tuple comprising state, observation, action space, and a reward function that balances energy efficiency with communication performance metrics such as throughput, packet loss ratio, and end-to-end delay. Evaluated through NS-3 simulations, the proposed model demonstrates robust performance, achieving approximately 90% B2 energy retention, throughput up to 0.14 Mbps, packet loss reduced to around 2%, and delay stabilized at about 0.06 s over a 500-slot episode. Comparative analysis and statistical validation underscore its superiority, establishing a scalable and effective solution for prolonged SAR operations in challenging environments.

## Data Availability

The datasets used and/or analysed during the current study are available from the corresponding author upon reasonable request.
